# Can patient-reported outcome measures predict mortality in neurological populations? A systematic review

**DOI:** 10.3389/fneur.2026.1705393

**Published:** 2026-01-28

**Authors:** Hyunjun Ahn, Yadi Li, Nicolas Thompson, LaDonna Pierce, Irene Katzan, Brittany Lapin

**Affiliations:** 1 Lerner College of Medicine, Cleveland Clinic, Cleveland, OH, United States; 2 Department of Quantitative Health Sciences, Cleveland Clinic, Cleveland, OH, United States; 3 Floyd D. Loop Alumni Library, Cleveland Clinic, Cleveland, OH, United States; 4 Cerebrovascular Center Neurological Institute, Cleveland, OH, United States

**Keywords:** mortality, neurology, patient reported outcomes, prediction model, quality of life, survival, systematic review

## Abstract

**Background:**

Patient-reported outcome measures (PROMs) are increasingly used for symptom monitoring and care delivery, yet their prognostic value for identifying patients at higher risk for mortality in neurological populations is unclear. This systematic review evaluated whether PROMs predict mortality and/or survival in adults with neurological conditions.

**Methods:**

We systematically searched MEDLINE, Embase, and the Cochrane Central Register of Controlled Trials (January 2002–November 2024) for studies incorporating PROMs into mortality or survival prediction models across 10 neurological conditions: motor neuron disease, diabetic neuropathy, nervous system cancers, Alzheimer’s and other dementias, Guillain–Barré syndrome, epilepsy, headache, multiple sclerosis, Parkinson’s disease, and stroke. Screening, data extraction, and risk-of-bias assessment followed the CHARMS and PRISMA guidelines. Findings were descriptively summarized.

**Results:**

Of 6,218 abstracts reviewed, 49 studies met the inclusion criteria. Most evaluated stroke (*n =* 16), nervous system cancers (*n =* 14), or motor neuron disease (*n =* 9). None evaluated headache, diabetic neuropathy, Guillain–Barré syndrome, or epilepsy. Of the included studies, 26 used generic PROMs, 19 used condition-specific PROMs, and 4 included both. Across conditions, PROMs independently predicted mortality in three-quarters of studies, with the strongest evidence observed in nervous system cancers and motor neuron disease. By instruments, EORTC QLQ in brain cancers and SF-36 in stroke showed the most consistent prognostic utility. Among studies with mixed findings by domain, physical health components were more likely to predict mortality than emotional components.

**Conclusion:**

PROMs independently predict mortality in several neurological conditions, though prognostic value varied by condition and instrument type. Future studies should evaluate their additive value and feasibility for integration into prognostic models in routine care.

## 1 Introduction

Neurological disorders represent a major global health burden, affecting an estimated 3.40 billion individuals in 2021 and contributing to approximately 11.1 million deaths annually (1). Collectively, they rank among the top contributors to global disability-adjusted life years and years of life lost, second only to cardiovascular diseases (1). Early identification of clinical decline and prognosis is critical for supporting patient-centered decision-making. Patient-reported outcomes (PROs), which capture patients’ subjective assessments of their health without clinician interpretation, are gaining recognition as valuable tools in this process (2–4).

Patient-reported outcome measures (PROMs) are increasingly integrated into routine clinical practice across various healthcare systems (5–7). They support diverse functions, including symptom screening, evaluating the impact of disease or treatment, auditing the quality of care, measuring healthcare utilization, and facilitating doctor–patient communication (4, 6, 8–12). In addition to these applications, PROMs have demonstrated prognostic value for hard clinical endpoints, such as mortality, in cardiology (13–16), oncology (17, 18), and other chronic illnesses (2). However, their prognostic value for mortality in neurological conditions remains less well established. For instance, a prior systematic review on the use of self-rated health (SRH) in stroke populations reported mixed findings regarding its association with survival (19). Furthermore, heterogeneity in PROM instruments and statistical analytical approaches across neurological studies presents challenges in evaluating their prognostic value.

The objective of this study was to evaluate the ability of PROMs to predict mortality and/or survival in neurological populations. The aims of our study were to systematically review published peer-reviewed articles describing predictive models for mortality and/or survival involving PROMs and to summarize their findings, including the types of PROMs used, the predictive ability of the PROMs, and the challenges and opportunities for informing the implementation of these emerging models in healthcare settings.

## 2 Methods

This review was registered in PROSPERO (CRD420250617247) and follows the recommendations of the Preferred Reporting Items for Systematic Reviews and Meta-Analyses (PRISMA) guidelines (20).

### 2.1 Data sources and searches

A comprehensive search strategy was developed in collaboration with a medical librarian to systematically search relevant literature in MEDLINE, Embase, and the Cochrane Central Register of Controlled Trials on 11 November 2024 (Appendix 1). Additionally, the reference lists of included studies were reviewed to identify any missed journal articles.

The search strategy focused on three primary concepts: (1) mortality and/or survival, (2) PROMs, and (3) predictive models. For mortality and/or survival, we included terms for in-hospital mortality, 30-day mortality, 90-day mortality, overall mortality, survival, recurrence-free survival, relapse-free survival, and/or progression-free survival. PROM-related terms encompassed patient- or self-reported outcomes and proxy-reported outcomes, including health-related quality of life, quality of life, health utility, and/or condition-specific measures. Finally, terms for predictive models included predictive models, algorithms, artificial intelligence, machine learning, informatics, risk prediction, statistical models, and/or clinical decision support.

### 2.2 Eligibility criteria

The criteria for including studies in this research were based on the following PICOTS framework: P (Population), I (Intervention), C (Comparator), O (Outcomes), T (Time), and S (Study Design).

#### 2.2.1 Population

Adult patients (≥18 years of age) with a neurological condition, as defined by the global burden of neurological disease, were included in this review (1). Infectious, traumatic, congenital, and developmental disorders were excluded. The review focused on the following 10 neurological conditions: (1) motor neuron disease (amyotrophic lateral sclerosis, primary lateral sclerosis, progressive bulbar palsy, progressive muscular atrophy), (2) diabetic neuropathy, (3) nervous system cancers (including brain tumors, peripheral nerve tumors, and spinal cord tumors), (4) Alzheimer’s and other dementias (vascular dementia, Alzheimer’s dementia, Lewy body dementia, frontotemporal dementia, and mild cognitive impairment), (5) Guillain–Barré syndrome, (6) epilepsy, (7) headache (migraine and tension-type headache), (8) multiple sclerosis, (9) Parkinson’s disease, and (10) stroke (ischemic, intracerebral hemorrhage, subarachnoid hemorrhage, and transient ischemic attack).

#### 2.2.2 Intervention/exposure

PROMs were considered the intervention or exposure of interest.

#### 2.2.3 Comparison

No specific comparator was required.

#### 2.2.4 Outcomes

Survival and/or mortality were the outcomes of interest.

#### 2.2.5 Time

A study was eligible for inclusion if it was published in English between 1 January 2002 and 8 November 2024, with an available full text. The 2002 start date was selected based on the increased adoption of PROMs following the publication of “Crossing the Quality Chasm: A New Health System for the 21st Century” by the Institute of Medicine Committee, which emphasized patient-centeredness as one of the six aims of quality healthcare (21). This led regulatory bodies such as the FDA and EMA to recognize PROMs, which were previously limited and infrequently used in healthcare, and to promote their integration into clinical research and healthcare delivery.

#### 2.2.6 Study design

Eligible studies were required to describe a model predicting mortality and/or survival by incorporating PROMs in patient populations with selected neurological conditions. All model types were considered, including Cox proportional hazards regression, logistic regression, random forests, and neural network architectures.

#### 2.2.7 Exclusion criteria

Studies focusing on individuals aged under 18 years were excluded.

### 2.3 Study selection and data extraction

Abstracts were screened using Covidence (Covidence systematic review software, Veritas Health Innovation, Melbourne, Australia) by two independent reviewers drawn from a pool of four reviewers (MA, YL, NT, and/or BL) (22). Conflicting decisions were evaluated and resolved by group consensus (MA, YL, NT, and BL). Full-text screening was then conducted similarly by two independent reviewers, with any conflicts resolved by group consensus.

Following full-text screening, the remaining publications were eligible for data extraction. A data extraction template was developed based on the Critical Appraisal and Data Extraction for Systematic Reviews of Prediction Modeling Studies (CHARMS) checklist (23), including source of data, participant description (including recruitment method), outcomes to be predicted, candidate predictors, sample sizes, missing data, model development (methods, assumptions, predictor selection, shrinkage), model performance (calibration, discrimination, classification measures), results (model coefficients), interpretation (including clinical relevance and future directions), and discussion of generalizability and limitations. Additional items were included to describe the PROM, its categorization, and the statistical analyses. The extraction sheet was revised to account for heterogeneity in study types, and comment boxes were included to provide an overall summary of the study findings (Supplementary Table 1).

Two independent reviewers extracted publication information into Covidence, with the second reviewer comparing extraction fields and finalizing consensus. Discrepancies were resolved by group consensus during weekly meetings. During extraction, relevant references were checked, and manuscripts were manually added to the full-text screening stage as applicable.

Risk of bias assessments were conducted similarly by two independent reviewers, with the second reviewer comparing extraction fields and determining consensus; any discrepancies were discussed and finalized during weekly group meetings. Risk of bias was based on an adapted version of the CHARMS checklist (23) (Supplementary Table 2).

### 2.4 Synthesis methods

Given the anticipated small number of eligible studies and substantial heterogeneity in how PROMs were measured and incorporated into prediction models, we summarized the results using descriptive statistics and narrative synthesis. We defined PROMs as condition-specific if they were developed to assess aspects of health specific to the disease, and generic if they were designed to assess general aspects of health across conditions (see Supplementary Table 3 for a full list of PROMs with classification).

The primary focus was not on effect measures but on whether the PROM was significantly associated with mortality or survival. When possible, the results were stratified by neurological condition to account for expected variability across disease groups.

## 3 Results

### 3.1 Overview of included articles

**Figure 1:**
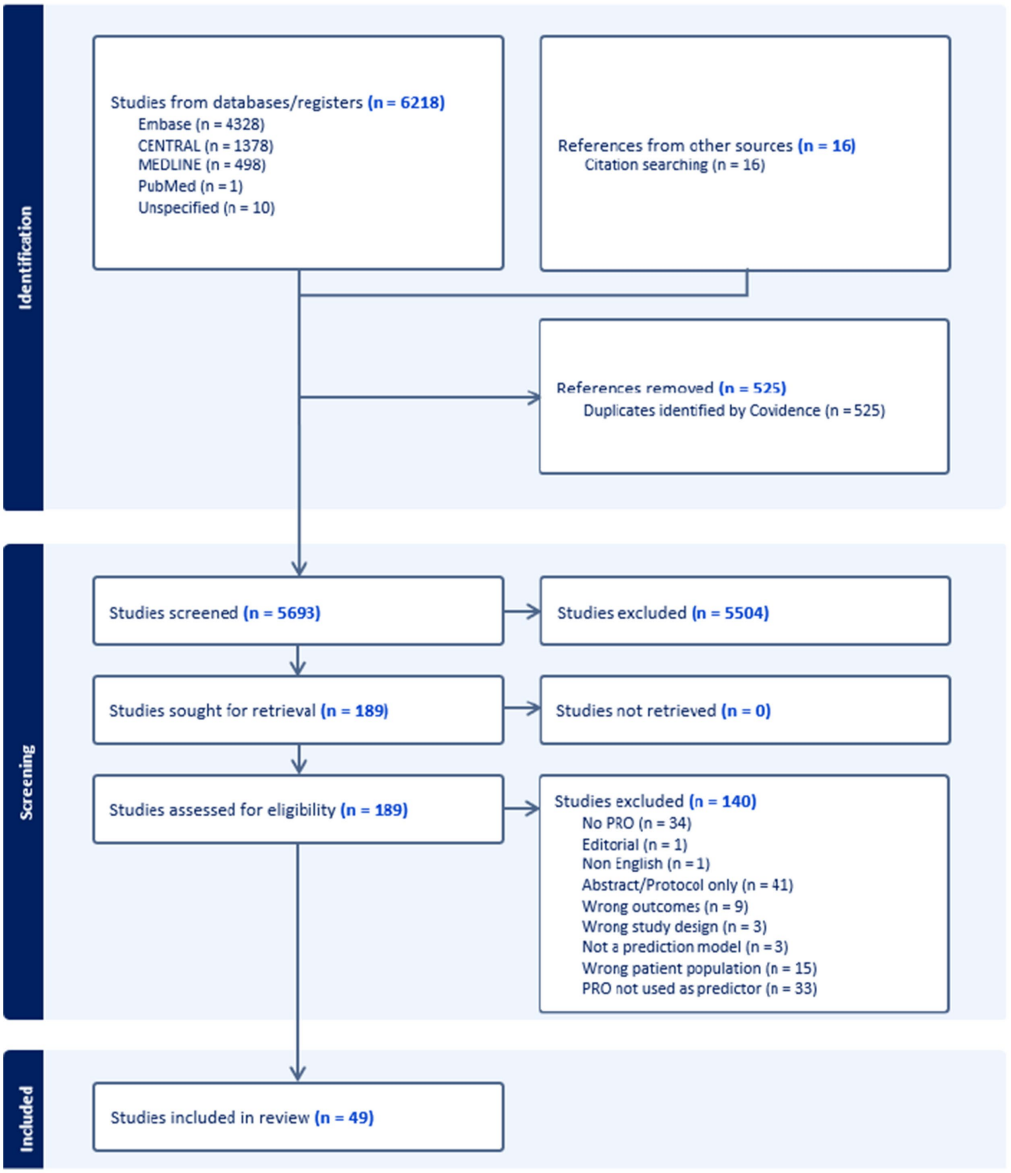
PRISMA flow diagram. PRISMA: Preferred Reporting Items for Systematic Reviews and Meta-Analyses.

Of the 6,218 identified studies, 525 studies were removed as duplicates. Title and abstract screening of the remaining 5,693 studies yielded 189 studies for full-text review, of which 49 met the inclusion criteria (Figure 1). The included studies spanned a broad range of geographic regions, with the majority conducted in the United States (*n =* 13), in the United Kingdom (*n =* 6), and across multiple countries (*n =* 6). The most frequently studied neurological conditions were stroke (*n =* 16), nervous system cancers (*n =* 14), and motor neuron disease (*n =* 9). The number of participants who completed PROMs in the included studies ranged from 57 to 3,667. Of the included studies, 30 were prospective cohort studies, 12 were randomized controlled trials (RCTs), 5 were retrospective cohort studies, and 1 was a systematic review. One study pooled individual patient data from six RCTs and two prospective cohort studies, all of which were assessed using the same methods (24).

### 3.2 Overview of PROMs administered

The characteristics of the PROMs used across studies are summarized in Tables 1, 2. Overall, 26 studies used generic instruments, 18 used condition-specific instruments, and 5 studies used both. The most commonly used generic instruments were the 36-Item Short-Form Survey (SF-36) (*n =* 8) and the SRH item (*n =* 6) (Supplementary Table 3). Among condition-specific instruments, the European Organization for Research and Treatment of Cancer Quality of Life Questionnaire and Brain Cancer Module (EORTC QLQ-C30/BN20) (*n =* 7), commonly used in brain cancer populations, and the Amyotrophic Lateral Sclerosis Functional Rating Scale—Revised (ALSFRS-R) (*n =* 4), commonly used in ALS, were the most frequently reported (Supplementary Table 3). PROMs were assessed at multiple time points in only 14 studies, and 7 studies evaluated the association between changes in PROMs over time and mortality.

**Table 1:** Summaries of included studies.

Study characteristics	Number	(%)	Reference(s)
Clinical conditions			
Nervous system cancer	14	(28.6)	(25, 26, 30, 39–41, 67–74)
Stroke	16	(32.7)	(19, 28, 31, 34, 46–56, 75)
Motor neuron disease	9	(18.4)	(37, 38, 45, 76–81)
Parkinson’s disease	3	(6.1)	(27, 29, 82)
Dementia	6	(12.2)	(24, 32, 33, 35, 36, 83)
Multiple sclerosis	1	(2.0)	(84)
Type of PROMs used			
Generic	26	(53.1)	(19, 24, 25, 28, 30–36, 46–56, 69, 70, 78, 83)
Condition-specific	19	(38.8)	(29, 37–41, 45, 67, 68, 71–76, 80–82, 84)
Both	4	(8.2)	(26, 27, 77, 79)
Univariable prediction of mortality by condition			
Yes	34	(69.4)	
Nervous system cancer	13	(26.5)	[25, 26, 30, 39–41, 63, 68–73]
Stroke	8	(16.3)	(28, 46, 47, 49–52, 56)
Motor neuron disease	7	(14.3)	(37, 38, 77–81)
Parkinson’s disease	3	(6.1)	(27, 29, 82)
Dementia	2	(4.1)	(32, 35)
Multiple sclerosis	1	(2.0)	(84)
No	4	(8.2)	
Stroke	1	(2.0)	(54)
Motor neuron disease	1	(2.0)	(45)
Dementia	2	(4.1)	(36, 83)
Not conducted	11	(22.4)	
Nervous system cancer	1	(2.0)	(68)
Stroke	7	(14.3)	(19, 31, 34, 48, 53, 55, 75)
Motor neuron disease	1	(2.0)	(76)
Dementia	2	(4.1)	(24, 32)
Multivariable prediction of mortality by conditions			
Yes (including those varying by model)*	33	(67.3)	
Nervous system cancer	11	(22.4)	(30, 39–41, 67–69, 71–74)
Stroke	10	(20.4)	(31, 34, 46–52, 56)
Motor neuron disease	6	(12.2)	(37, 76–79, 81)
Parkinson’s disease	1	(2.0)	(82)
Dementia	4	(8.2)	(24, 32, 33, 35)
Multiple sclerosis	1	(2.0)	(84)
No	10	(20.4)	
Nervous system cancer	2	(4.1)	(25, 26)
Stroke	4	(8.2)	(28, 53–55)
Motor neuron disease	1	(2.0)	(45)
Dementia	1	(2.0)	(36)
Parkinson’s disease	2	(4.1)	(27, 29)
Not conducted	6	(12.2)	
Nervous system cancer	1	(2.0)	(70)
Stroke	2	(4.1)	(19, 75)
Motor neuron disease	2	(4.1)	(38, 80)
Dementia	1	(2.0)	(83)
Univariable prediction of mortality by type of PROMs			
Yes^a^	36	(73.5)	
Generic	18	(36.7)	(25–28, 30, 32, 35, 46, 47, 49–52, 56, 69, 70, 78, 79)
Condition-specific	18	(36.7)	(27, 29, 37–41, 67, 71–74, 77, 79–82, 84)
No^b^	5	(10.2)	
Generic	3	(6.1)	(36, 54, 83)
Condition-specific	2	(4.1)	(26, 45)
Not conducted^c^	12	(24.5)	
Generic	9	(18.4)	(19, 24, 31, 33, 34, 48, 53, 55, 77)
Condition-specific	3	(6.1)	(68, 75, 76)
Multivariable prediction of mortality by type of PROMs			
Yes (including those varying by model)^d^*	35	(71.4)	
Generic	19	(38.8)	(24, 30–35, 46–52, 56, 69, 77–79)
Condition-specific	16	(32.7)	(37, 39–41, 67, 68, 71–74, 76, 77, 79, 81, 82, 84)
No^e^	12	(24.5)	
Generic	8	(16.3)	(25–28, 36, 53–55)
Condition-specific	4	(8.2)	(26, 27, 29, 45)
Not conducted	6	(12.2)	
Generic	3	(6.1)	(19, 70, 83)
Condition-specific	3	(6.1)	(38, 75, 80)

^a–d^Four studies used both generic and condition-specific instruments (26, 27, 77, 79). ^a^Two studies found univariable significance in both instrument types (27, 79). ^b^One study found univariable significance for the generic but not the condition-specific instrument (26). ^c^One study conducted univariable analysis for the condition-specific but not the generic instrument (77). ^d^Two studies found significant associations for both instrument types (77, 79). ^e^Two studies found no significant associations for either type of instrument (26, 27). *Three studies reported variations in the significance by model (30–32).

**Table 2:** Study characteristics.

Study	Country	Study design	Patient condition	Number of participants with completed PROs	Age*	Type of PROM	Instrument(s)	Timing of assessment	Outcome definition (timeframe)
Ackrivo et al. (37)	USA	Retrospective cohort study	Amyotrophic lateral sclerosis	765	63	Condition-specific	ALSFRS-R	Baseline	Respiratory insufficiency, defined as initiation of NIV, FVC < 50% of predicted, tracheostomy placement, or death (6 months and median 2.3 years, ≤10.7 years)
Araujo et al. (85)	Brazil	Systemic review	Stroke	Variable; sample size ranged from 19 to 104,876	Variable	Generic	SRH	Variable	Variable
Armstrong et al. (67)	USA	Randomized controlled trial	Glioblastoma multiforme with a supratentorial component	91–172	58**	Condition-specific	EORTC QLQ-C30/BN20	Baseline and fourth cycle of chemotherapy	Survival interval (not reported)
Ayerbe et al. (34)	UK	Prospective cohort study	First-ever stroke	1,354	62.4% were >64	Generic	HADS	3-month follow-up after first-ever stroke	Mortality (5 years)
Bell et al. (46)	USA	Prospective cohort study	Stroke	3,143	72.6	Generic	SF-36 physical functioning, CES-D, MOS social support	Last measure before stroke	Mortality (≤17 years)
Bosma et al. (25)	Netherlands	Prospective cohort study	WHO grade III–IV glioma (astrocytoma, oligodendroglioma, or oligoastrocytoma)	68	54.4	Generic	SF-36 mental component summary	Baseline and every 4 months until 16 months after diagnosis	Mortality (16 months)
Brown et al. (26)	USA	Randomized controlled trial	Anaplastic astrocytoma or glioblastoma	107–116 for the first follow-up; 72–75 for the second follow-up	54	Generic + Condition-specific	LASA, FACT-Br, SDS-fatigue, ESS, POMS-SF fatigue, POMS-SF depression	≤72 h post-entry, 2 months, and 4 months	Overall survival (not reported)
Chio et al. (76)	Italy	Prospective cohort study	Amyotrophic lateral sclerosis	128	64.7	Condition-specific	FrSBe	Within 4 months of ALS diagnosis	Tracheostomy-free survival (≤4.5 years)
DelAguila et al. (77)	USA	Prospective cohort study	Amyotrophic lateral sclerosis	≥139 for SF-36; 155 for ALS severity score	61.3	Generic + Condition-specific	SF-36 and ALS severity score	Invited after diagnosis (mean 5.4 months, median 4 months)	Tracheostomy-free survival (≤9.67 years)
Ediebah et al. (68)	Belgium, Austria, Finland, France, Germany, Hungary, Italy, Netherlands, Portugal, Sweden, Switzerland, UK (International)	Randomized controlled trial	Anaplastic oligodendroglioma or oligoastrocytoma	288	48–49**	Condition-specific	EORTC QLQ-C30/BN20	Baseline, end of radiotherapy, every 3 months for the first year after radiotherapy, then every 6 months until recurrence of the disease	Overall survival (median f/u 140 months)
Ertel et al. (53)	USA	Randomized controlled trial	Stroke	269	69.7	Generic	CES-D	Baseline	Mortality (mean 47 months)
Forsaa et al. (82)	Norway	Prospective cohort study	Parkinson’s disease	230	64.9	Condition-specific	Stavanger Sleepiness Questionnaire REM sleep behavior disorder subscore and UPDRS I psychosis subscore	Baseline visit and annually until 2005	Mortality (≤12 years)
Glader et al. (51)	Sweden	Prospective cohort study	Stroke	3,667	71.8	Generic	Self-perceived fatigue and dependency on primary ADL	2-year follow-up	Mortality (3 years)
Grool et al. (31)	Netherlands	Prospective cohort study	Peripheral arterial disease, coronary artery disease, cerebrovascular disease, or abdominal aortic aneurysm	1,110	59	Generic	SF-36 mental component summary	Baseline	Mortality (≤9 years)
Hubbard et al. (47)	Australia	Prospective cohort study	Stroke	931	72.7	Generic	SF-36 physical functioning	Every 3 years between 1999 and 2011	Mortality (≤14.4 years)
Krishnan et al. (29)	India	Retrospective cohort study	Advanced Parkinson’s disease with bilateral STN deep brain stimulation	Unclear	56.5	Condition-specific	UPDRS II	Baseline	Dementia-free survival (mean 4.7 years, ≤12 years)
Liira et al. (24)	Finland	6 RCTs, 2 prospective cohort studies	Dementia	214	77	Generic	15D	Baseline	Mortality (2 years)
Lou et al. (78)	USA	Randomized controlled trial	Amyotrophic lateral sclerosis	268	Not reported	Generic	MQoL-SIS	Baseline and monthly evaluations for 13 months	Mortality (5 months)
Mauer et al. (40)	Belgium, Germany, Spain, France, Italy, Netherlands, Poland, Israel, Sweden, Slovenia, United Kingdom, Austria, Canada, Switzerland, Australia (International)	Randomized controlled trial	Glioblastoma	490	70% were ≥50	Condition-specific	EORTC QLQ-C30/BN20	Baseline: <1 month before or <2 weeks after randomization	Overall survival (not reported)
Mauer et al. (41)	Belgium, Austria, Finland, France, Germany, Hungary, Italy, Netherlands, Portugal, Sweden, Switzerland, UK (International)	Randomized controlled trial	Anaplastic oligodendroglioma or oligoastrocytoma	247	31% were ≥54	Condition-specific	EORTC QLQ-C30/BN20	Baseline: <6 weeks before or after randomization	Overall survival (not reported)
McCarter et al. (30)	Canada	Prospective cohort study	Primary brain tumors	93	52	Generic	HUI	Baseline after diagnosis but before treatment	Overall survival (5 years)
Mead et al. (48)	UK	Randomized controlled trial	Ischemic stroke	1,006 for SF-36; unclear for others	71.1	Generic	SF-36 (excluding role-physical)	Variable; average 64 months after stroke	Overall survival (mean 5.2 years, ≤7.67 years)
Nielsen et al. (36)	Denmark	Randomized controlled trial	Mild Alzheimer’s with MMSE > = 20	321	76.2	Generic	SRH	Baseline	Mortality (3 years)
Noll et al. (69)	USA	Retrospective cohort study	Supratentorial glioblastoma	102	~50.9	Generic	BDI-II	Within 1 month of surgery (mean 23.9 days, SD 11.0)	Overall survival (≤ 21 years)
Paillisse et al. (38)	France	Prospective cohort study	Amyotrophic lateral sclerosis treated with Riluzole	Unclear (~1,550)	62.3	Condition-specific	VAS for cramps, stiffness, tiredness, and fasciculations	Baseline and every 3 months	Overall survival (≤ ~ 21.37 months***)
Paquette et al. (39)	France	Prospective cohort study	Supratentorial glioblastoma	102	59.6	Condition-specific	EORTC QLQ-C30/BN20	Baseline	Overall survival (median 24 months)
Peters et al. (73)	USA	Prospective cohort study	Recurrent WHO grade II–IV glioma	237	50	Condition-specific	FACT-G, FACT-Br, and FACIT-F	Variable; any stage after diagnosis of recurrent disease	Overall survival (median 27.60 months; ≤38 months)
Phung et al. (35)	Denmark	Randomized controlled trial	Dementia	328 for self-rated and 329 for caregiver-rated EQ-VAS	82.7% were ≥70	Generic	EQ-VAS by self- and caregiver-rated health	Baseline	Mortality (3 years)
Quinten et al. (74)	Belgium (International)	Randomized controlled trial	Brain cancer	829	23% were >60	Condition-specific	EORTC QLQ-C30	Baseline	Overall survival (≤ ~ 98 months***)
Mokhber et al. (55)	Iran	Prospective cohort study	First-ever stroke	624	64.7	Generic	Self-reported acute psychological stress questionnaire	Within 2 weeks of stroke	Mortality (3 months, 1 year, and 5 years)
Sturm et al. (52)	Australia	Prospective cohort study	First-ever stroke	93; 15 (16%) assessed by proxy	72	Generic	AqoL	3 months follow-up	Mortality (12 months)
Thakore et al. (45)	USA	Retrospective cohort study	Amyotrophic lateral sclerosis	3,199	57**	Condition-specific	ALSFRS-R pre-slope	Throughout the RCT	Staging through disease progression to mortality (not reported)
Thakore et al. (79)	USA	Retrospective cohort study	Amyotrophic lateral sclerosis	964 for PHQ-9 and 673 for ALSFRS-R	60.6	Generic + Condition-specific	PHQ-9, ALSFRS-R, and ALSFRS-R pre-slope	Initial and at follow-up appointments	Overall survival (≤ ~ 9 years***)
vanEijk et al. (80)	Netherlands	Prospective cohort study	Amyotrophic lateral sclerosis, progressive muscular atrophy, or primary lateral sclerosis	423	65	Condition-specific	ALSFRS-R	Baseline and after diagnosis	Overall survival (≤2.4 years)
Wang et al. (27)	China	Prospective cohort study	Parkinson’s disease	218	56.9	Generic + Condition-specific	FSS and UPDRS II Dysphagia subscore	Baseline	Overall survival (mean 9.58 years, median 10.31 years, ≤11.65 years)
Winovich et al. (54)	USA	Prospective cohort study	Stroke	717	81.0	Generic	CES-D	Before stroke; included if measured ≤5 years before incident stroke	Overall survival (≤22 years)
Xu et al. (81)	Australia	Prospective cohort study	Amyotrophic lateral sclerosis	84 for ALS-FTD-Q and 66 for MiND-B	63.8	Condition-specific	ALS-FTD-Q and MiND-B	Not stated	Overall survival (≤ ~ 15 years***)
Zietemann et al. (75)	Germany; France	Prospective cohort study	Stroke	274	67.0	Condition-specific	IQCODE	Baseline	Mortality (3 years)
John et al. (70)	USA	Prospective cohort study	Primary brain tumors	60	51	Generic	BDI-II	Pre-treatment evaluation	Overall survival (≤ ~ 28 months***)
Wefel et al. (71)	USA, Canada, and Israel (International)	Randomized controlled trial	Glioblastoma multiforme	485 (95%) completed at baseline, >75% completion through 46 weeks	~58**	Condition-specific	EORTC QLQ-C30/BN20 and MDASI-BT	Baseline; end of radiation (week 6); during treatment (weeks 10, 22, 34, 46)	Overall survival (≤ ~ 30 months***)
Mavaddat et al. (28)	UK	Prospective cohort study	First-ever stroke	776	76.2	Generic	SRH	Baseline	Mortality (2 years and 13 years)
Xie et al. (83)	UK	Prospective cohort study	Dementia	417	84**	Generic	SRH	Latest measure before the estimated onset of dementia	Overall survival (14 years)
Hillen et al. (56)	UK	Prospective cohort study	First-ever stroke	561 at initial SRH; 356 (81.7% of eligible participants) at 1-year follow-up	69.4	Generic	SRH and SRH transition	Baseline and follow-up	Recurrence-free survival (1 year and 5 years)
González-Vélez et al. (32)	Spain	Prospective cohort study	Dementia	525 assessed by proxy	85.6	Generic	EQ-5D	Baseline	Mortality (18 months)
Walker et al. (33)	Canada	Prospective cohort study	Cognitive impairment	1,081 in mild–moderate cognitive impairment, 469 in severe cognitive impairment group	75.7	Generic	SRH	Baseline	Overall survival (Mean: 5 years, ≤5.67 years)
Sehlen et al. (72)	Germany	Prospective cohort study	Malignant intracranial neoplasma	57	~51.5**	Condition-specific	FACT-G	Baseline	Overall survival (≤ ~ 88 months***)
Raffel et al. (84)	UK	Prospective cohort study	Multiple sclerosis	2,126 for MSIS-29 Physical, 2,119 for MSIS-29 Psychological, 625 for prEDSS	54.0	Condition-specific	MSIS-29 physical and psychological score	Baseline and 1-year follow-up	Mortality (10 years)
Kielbergerová et al. (50)	Czech Republic	Prospective cohort study	First-ever stroke	341	69.0	Generic	SF-36 and HADS	< 6 months from stroke (median 19 months)	Mortality (5 years)
Naess and Nyland (49)	Norway	Prospective cohort study	First-ever stroke	188	48	Generic	SF-36 and NHP-I	Variable; average 6 years after stroke	Mortality (mean 12.4 years)

15D, 15-dimensional instrument; ADL, Activities of Daily Living; ALSFRS-R, Revised Amyotrophic Lateral Sclerosis Functional Rating Scale; ALS-FTD-Q, Amyotrophic Lateral Sclerosis—Frontotemporal Dementia Questionnaire; AQoL, Assessment of Quality of Life; BDI, Beck Depression Index; CES-D, Center for Epidemiologic Studies Depression Scale; EORTC, QLQ-BN20 European Organization for Research and Treatment of Cancer Brain Cancer Module; EORTC, QLQ-C30 European Organization for Research and Treatment of Cancer Quality of Life Questionnaire; EQ-5D, EuroQoL five dimensions questionnaire; EQ-VAS, EuroQol Visual Analogue Scale; ESS, Epworth Sleepiness Scale; FACIT-F, Functional Assessment of Chronic Illness Therapy—Fatigue; FACT-Br, Functional Assessment of Cancer Therapy—Brain; FACT-G, Functional Assessment of Cancer Therapy—General; FrSBe, Frontal Systems Behavior Scale; FSS, Fatigue Severity Scale; HADS, Hospital Anxiety and Depression Scale; HUI, Health Utilities Index; IQCODE, Informant Questionnaire on Cognitive Decline in the Elderly; LASA, Linear Analog Scale Assessment; MDASI-BT, MD Anderson Symptom Inventory—Brain Tumor; MiND-B, Motor Neuron Disease Behavioral Instrument; MOS, Medical Outcomes Study; MQOL-SIS McGill Quality of Life Questionnaire Single-Item Scale; MSIS, Multiple Sclerosis Impact Scale; PHQ, Patient Health Questionnaire; POMS-SF Short Form of the Profile of Mood States; SDS, Symptom Distress Scale; SF-36, 36-Item Short-Form Survey; SRH self-rated health; UPDRS, Unified Parkinson’s Disease Rating Scale; VAS, Visual Analogue Scale; NHP-I, Nottingham Health Profile. *Mean in years unless otherwise specified **Median in years ***Years estimated from the survival curve.

### 3.3 Aims and methods

A total of 6 (12.2%) studies aimed to develop a prediction model, and 43 (87.8%) examined the association between PROMs and survival without the intent to develop a prediction model. Of the six studies that aimed to develop a prediction model, five specifically aimed to address whether PROMs could predict mortality. Most studies (*n =* 44) used Cox proportional hazards regression. In contrast, four studies used logistic regression, two studies used a Weibull survival model, and one study used Markov multistate modeling (Table 3).

**Table 3:** Statistical analyses and conclusions regarding PROMs.

Study	Patient condition	Instrument(s)^a^	Statistical analysis	Aimed to develop a prediction model? (Y/N)	Prediction of mortality in the univariable model? (Y/N)	Comments^b^	Prediction of mortality in a multivariable model? (Y/N)	Comments^b^	Conclusion about PROMs
Ackrivo et al. (37)	Amyotrophic lateral sclerosis	ALSFRS-R	Logistic regression	Y	Y		Y		ALSFRS-R scores were significantly associated with mortality. Applying the prognostic model may help guide the optimal timing of referrals for patients with ALS, with the aim of delaying the onset of respiratory insufficiency
Araujo et al. (85)	Stroke	SRH	Variable; Cox proportional hazards (in studies using SRH to predict mortality)	N	N/A		N/A		Systematic review: The predictive value of SRH for stroke mortality remains inconclusive, with two studies reporting significant associations and two reporting none. Nevertheless, routine assessment of SRH may aid in summarizing overall health status, identifying high-priority patients, and monitoring individuals at risk for stroke
Armstrong et al. (67)	Glioblastoma multiforme with a supratentorial component	EORTC QLQ-C30/BN20	Cox proportional hazards	N	Y		Y	EORTC QLQ-C30: baseline physical functioning scale and nausea/vomiting item. EORTC QLQ-BN20: early changes in the cognitive functioning scale, motor dysfunction item, and hair loss item	Baseline and early changes in net clinical benefit, measured by EORTC QLQ-C30/BN20, were associated with lower rates of survival
Ayerbe et al. (34)	First-ever stroke	HADS	Cox proportional hazards	N	N/A		Y		Depression was associated with increased long-term mortality following stroke, with a stronger association observed among younger stroke survivors (<65 years old)
Bell et al. (46)	Stroke	SF-36 physical functioning, CES-D, MOS social support	Cox proportional hazards	N	Y	SF-36 physical function	Y	SF-36 physical function	Poor pre-stroke physical function was associated with increased post-stroke mortality, alongside other contributing factors
Bosma et al. (25)	WHO grade III–IV glioma (astrocytoma, oligodendroglioma, or oligoastrocytoma)	SF-36 mental component summary	Cox proportional hazards	N	Y		N		Higher mental functioning after surgery was associated with longer survival, but did not provide additional independent predictive value beyond established patient and tumor characteristics
Brown et al. (26)	Anaplastic astrocytoma or glioblastoma	LASA, FACT-Br, SDS-fatigue, ESS, POMS-SF fatigue, POMS-SF depression	Cox proportional hazards	N	Y	Baseline POMS depression at first and second follow-up, and decline in POMS depression between baseline and the first follow-up	N		Baseline QoL measures were predictive of impaired QoL over time, but were not independently associated with survival
Chio et al. (76)	Amyotrophic lateral sclerosis	FrSBe	Cox proportional hazards	N	N/A		Y	Both the FrSBe total scores and FrSBe executive dysfunction subscales	Neurobehavioral dysfunction and isolated dysexecutive behavior in ALS were associated with reduced survival. Cognitive and behavioral assessments should be incorporated into ALS diagnostic evaluations and clinical trial eligibility screening
DelAguila et al. (77)	Amyotrophic lateral sclerosis	SF-36 and ALS severity score	Cox proportional hazards	N	Y	ALS severity score; N/A for SF-36	Y	ALS severity score, and SF-36 physical health summary	Physical health, and not mental health, summaries measured by the SF-36 and the ALS severity score were independently associated with survival
Ediebah et al. (68)	Anaplastic oligodendroglioma or oligoastrocytoma	EORTC QLQ-C30/BN20	Joint model (Cox proportional hazards and linear mixed effects model)	N	N/A		Y	Appetite loss	HRQoL was associated with survival, and part of a treatment’s survival benefit may be offset by its negative impact on HRQoL. Joint modeling with longitudinal PROs allows for less biased estimation of overall treatment efficacy
Ertel et al. (53)	Stroke	CES-D	Cox proportional hazards	N	N/A		N		Psychosocial interventions had a non-significant trend toward reduced mortality in stroke patients with fewer depressive symptoms, but showed a non-significant increase in mortality in those with more depressive symptoms. This may reflect greater engagement and adaptability among patients with better physical, cognitive, and emotional health
Forsaa et al. (82)	Parkinson’s disease	Stavanger Sleepiness Questionnaire REM sleep behavior disorder subscore and UPDRS I psychosis subscore	Cox proportional hazards	N	Y		Y	UPDRS I psychosis subscore	Psychotic symptoms independently predicted increased mortality among patients with Parkinson’s disease
Glader et al. (51)	Stroke	Self-perceived fatigue and dependency on primary ADL	Cox proportional hazards	N	Y		Y		Fatigue is an important, independent predictor of death in patients who suffered a stroke, even after adjusting for other important prognostic factors
Grool et al. (31)	Peripheral arterial disease, coronary artery disease, cerebrovascular disease, or abdominal aortic aneurysm	SF-36 mental component summary	Cox proportional hazards	N	N/A		Varies by model	Associations between mood problems and mortality became significant when stratified by the presence of a lacunar infarct	Mood problems were associated with worse prognosis in patients with symptomatic atherosclerotic disease, with particularly high mortality risk in patients with lacunar infarcts, but not in patients with white matter lesions. Patients with lacunar infarcts and mood problems may be especially vulnerable and warrant closer monitoring
Hubbard et al. (47)	Stroke	SF-36 physical functioning	Cox proportional hazards	N	Y		Y		Among women with stroke, poor physical function was associated with significantly higher mortality compared to adequate physical function, although most patients survived beyond 10 years. Programs to maintain physical function may be beneficial
Krishnan et al. (29)	Advanced Parkinson’s disease with bilateral STN deep brain stimulation	UPDRS II	Cox proportional hazards	N	Y	On drug ON state only	N		Limitation of ADL, measured by USPDR II, was not independently predictive of dementia-free survival
Liira et al. (24)	Dementia	15D	Cox proportional hazards	N	N/A		Y		The 15D generic quality-of-life measure predicted 2-year mortality independent of disease burden. It may not only assess HRQoL in older adults but also capture disability and health factors associated with poor prognosis. However, in older individuals with psychosocial impairments (e.g., caregiver burden or loneliness), additional measures of emotional and social wellbeing should be explored
Lou et al. (78)	Amyotrophic lateral sclerosis	MQoL-SIS	Cox proportional hazards	N	Y		Y	Baseline MQoL-SIS and MQoL-SIS slope	ALS patients with better baseline QoL or slower decline in QoL had better survival rates
Mauer et al. (40)	Glioblastoma	EORTC QLQ-C30/BN20	Cox proportional hazards	Y	Y	EORTC QLQ-C30 cognitive functioning, fatigue, physical functioning, global health status; EORTC QLQ-BN20 communication deficit, motor dysfunction, seizures, weakness of legs	Y	EORTC QLQ-C30 cognitive functioning, global health status, social functioning	Traditional analyses suggest that HRQoL is prognostic of mortality; however, more detailed evaluation revealed limited reliability, as indicated by minimal improvement in C-index and R^2^ when HRQoL scores were added to the clinical factors in the model
Mauer et al. (41)	Anaplastic oligodendroglioma or oligoastrocytoma	EORTC QLQ-C30/BN20	Cox proportional hazards	Y	Y	EORTC QLQ-C30 emotional functioning; EORTC QLQ-BN20 communication deficit, weakness of legs	Y	EORTC QLQ-C30 emotional functioning; EORTC QLQ-BN20 communication deficit, future uncertainty, weakness of legs	Traditional analyses suggest that baseline HRQoL is prognostic of mortality; however, a more detailed evaluation revealed that they may be limited, as indicated by minimal improvement in C-index and R^2^ when HRQoL scores were added to the clinical factors in the model. The prognostic value of changes from baseline HRQoL should be investigated
McCarter et al. (30)	Primary brain tumors	HUI	Proportional hazards model with Weibull distribution	N	Y	HUI2 self-care in low-grade tumors; HUI3 speech and dexterity in high-grade tumors	Varies by model		HRQoL scores can predict survival in adults with brain tumors, supporting the use of patient-reported health measures alongside standard clinical factors in clinical trials and routine cancer care
Mead et al. (48)	Acute ischemic stroke	SF-36 (excluding role-physical)	Cox proportional hazards	N	N/A		Y	Varies by model	Higher post-stroke fatigue, measured by SF-36 vitality, was associated with reduced survival. Further research on the natural history of fatigue before and after stroke is needed to clarify its role as an independent predictor. Nonetheless, interventions targeting post-stroke fatigue may be warranted and should be evaluated in clinical trials
Nielsen et al. (36)	Mild Alzheimer’s with MMSE ≥20	SRH	Cox proportional hazards	N	N		N		In older adult patients with mild Alzheimer’s disease, SRH was not associated with increased mortality risk. SRH may inadequately reflect disease severity due to cognitive decline affecting self-assessment. Proxy-rated health measures should be considered in this population
Noll et al. (69)	Supratentorial glioblastoma	BDI-II	Cox proportional hazards	N	Y		Y		Depressive symptoms and impaired executive function are independently associated with shorter overall survival in patients with GBM. Routine assessment of mood and cognition after surgery may offer valuable prognostic information and support targeted rehabilitative interventions
Paillisse et al. (38)	Amyotrophic lateral sclerosis treated with riluzole	VAS for cramps, stiffness, tiredness, and fasciculations	Cox proportional hazards, Weibull survival model	Y	Y	VAS tiredness	N/A	Final multivariable model did not include any PROs	Exploratory; no conclusions were made about PROs
Paquette et al. (39)	Supratentorial glioblastoma	EORTC QLQ-C30/BN20	Cox proportional hazards	Y	Y	QLQ-C30 emotional functioning, pain, financial difficulties; QLQ-BN20 future uncertainty, visual disorder, itchy skin	Y	Varies by model; final multivariable model: QLQ-BN20 future uncertainty	Baseline HRQoL had prognostic value in patients with UGB, supporting its use in risk stratification and suggesting that it could be helpful in designing future clinical trials
Peters et al. (73)	Recurrent WHO grade II–IV glioma	FACT-G, FACT-Br, and FACIT-F	Cox proportional hazards	N	Y	FACT-Br Brain cancer subscale, FACIT-Fatigue subscale	Y	FACIT-fatigue subscale	Fatigue was a strong independent predictor of survival in patients with recurrent high-grade glioma, improving prognostic accuracy when added to traditional factors. Routine assessment of fatigue is recommended, and interventions to manage fatigue warrant further investigation
Phung et al. (35)	Dementia	EQ-VAS by self- and caregiver-rated health	Cox proportional hazards	N	Y		Y	Caregiver-rated EQ-VAS	Self- and caregiver-rated health, as measured by the EQ-VAS, diverged in the very early stages of the disease, with patients rating their health higher than caregivers. Caregiver-rated EQ-VAS, and not self-rated, independently predicted patient mortality
Quinten et al. (74)	Brain cancer	EORTC QLQ-C30	Cox proportional hazards	N	Y	QLQ-C30 variables not specified	Y	EORTC QLQ-C30 cognitive functioning	HRQoL scales provide complementary prognostic value beyond standard clinical variables by capturing additional dimensions of patient wellbeing, feelings, and functioning, offering a more comprehensive understanding of the burden of cancer and its treatment
Mokhber et al. (55)	Acute, first-ever stroke	Self-reported acute psychological stress questionnaire	Logistic regression	N	N/A		N		Acute stress within 2 weeks before stroke was not associated with the severity and outcomes of stroke
Sturm et al. (52)	Acute, first-ever stroke	AQoL	Logistic regression	N	Y		Y		The AQoL is a valid and sensitive instrument for assessing health-related quality of life after stroke
Thakore et al. (45)	Amyotrophic lateral sclerosis	ALSFRS-R pre-slope	Markov multistate models	N	N	Analysis stratified by each ALS stages	N		The established prognostic value of ALSFRS-R decline in survival may be mediated by more rapid progression to subsequent ALS stages. However, the rate of decline was not independently associated with an increased risk of death within a given stage
Thakore et al. (79)	Amyotrophic lateral sclerosis	PHQ-9, ALSFRS-R and ALSFRS-R pre-slope	Cox proportional hazards	N	Y		Y		PHQ-9 predicted poorer survival after controlling for multiple recognized predictors of survival in patients with ALS. Appropriate screening and early intervention in vulnerable subgroups may be warranted
vanEijk et al. (80)	Amyotrophic lateral sclerosis, progressive muscular atrophy, or primary lateral sclerosis	ALSFRS-R	Cox proportional hazards	N	Y		N/A		Patient-reported preferences may offer unique prognostic information, providing a better reflection of individual disease severity and patient-perceived disease impact
Wang et al. (27)	Parkinson’s disease	FSS and UPDRS II Dysphagia subscore	Cox proportional hazards	N	Y		N		Fatigue may contribute to increased mortality risk in patients with PD, although the sample size may have been insufficient to detect its significance as an independent predictor in multivariable analysis, likely due to a weak effect
Winovich et al. (54)	Acute stroke	CES-D	Cox proportional hazards	N	N		N		Exploratory analysis evaluating the effect of pre-stroke measures of frailty on outcomes; no conclusions were drawn about PROMs
Xu et al. (81)	Amyotrophic lateral sclerosis	ALS-FTD-Q and MiND-B	Cox proportional hazards	N	Y	ALS-FTD-Q	Y	ALS-FTD-Q	The presence of behavioral impairment, measured by the ALS-FTD-Q but not the MiND-B (which assesses stereotypical behavior, apathy, and disinhibition), was associated with worse prognosis and may serve as a potential prognostic biomarker in ALS alongside cognitive assessments
Zietemann et al. (75)	Acute stroke	IQCODE	Cox proportional hazards	Y	N/A		N/A		No conclusions regarding IQCODE and mortality were reported; it was used as an exclusion criterion and as a covariate in mortality modeling, but specific results were not provided
John et al. (70)	Primary brain tumors	BDI-II	Cox proportional hazards	N	Y		N/A		In patients with recurrent malignant gliomas, depression was associated with shorter survival. Early recognition and treatment of depressive symptoms may improve glioma-associated survival, though further research is warranted
Wefel et al. (71)	Glioblastoma multiforme	EORTC QLQ-C30/BN20 and MDASI-BT	Cox proportional hazards	N	Y	Early changes in MDASI-BT symptom burden, interference, symptom inference—activities, symptom inference—mood, affective factor, cognitive factor, generalized; early changes in EORTC QLQ-C30/BN20 social functioning, motor dysfunction, communication deficit	Y	Baseline MDASI-BT neurologic factor, EORTC QLQ-C30/BN20 physical function; early changes in EORTC QLQ-C30/BN20 communication deficit, MDASI-BT cognitive factor	Baseline and early worsening of select HRQoL measures were predictive of both progression-free and overall survival, supporting their potential use as stratification factors or entry criteria in clinical trials. The development of predictive models incorporating PROs, among others, to identify patients likely to experience early benefit from bevacizumab may be of value. However, these findings should be interpreted with caution, as no correction for multiple comparisons was applied due to the exploratory nature of the study
Mavaddat et al. (28)	First-ever stroke	SRH	Cox proportional hazards	N	Y	SRH dichotomized to “excellent/good” *vs*. “fair/poor” for 2-year mortality	N		Although SRH predicts mortality in older adults without a history of stroke, it does not predict mortality in those with prior stroke. However, SRH may still hold prognostic value for identifying individuals at risk in those without a history of stroke
Xie et al. (83)	Dementia	SRH	Cox proportional hazards	N	N		N/A		Self-reported health was not associated with survival in patients with dementia
Hillen et al. (56)	First-ever stroke	SRH and SRH transition	Cox proportional hazards	N	Y	SRH transition	Y	SRH transition	A single questionnaire to assess changes in SRH, rather than baseline SRH, predicted mortality. Changes in SRH may better capture deterioration and recovery, with fewer measurement errors compared with repeated longitudinal assessments using the same instrument
González-Vélez et al. (32)	Dementia	EQ-5D	Logistic regression	N	Y		Varies by model	EQ-5D interacted with the Cornell Depression Scale, significant only for the Cornell Depression Scale <6	The effect of QoL on mortality in residents with dementia varied by depression status, with a stronger effect in those without depression, highlighting that QoL in long-term care settings is influenced by multiple factors. QoL assessments in this population should account for depression status. Additionally, efforts to improve QoL should be accompanied by disability rehabilitation interventions
Walker et al. (33)	Cognitive impairment	SRH	Cox proportional hazards	N	n/a	Univariable analysis done only for the entire population (including the cognitively intact group)	Y	Only the mild-to-moderate cognitive impairment group	Although SRH is a valid and independent measure of health and a predictor of mortality in patients with mild-to-moderate cognitive impairment (e.g., early dementia), its predictive value declines with greater cognitive deterioration
Sehlen et al. (72)	Malignant intracranial neoplasma	FACT-G	Cox proportional hazards	N	Y		Y		HRQoL may help estimate a patient’s survival time. Further research is needed to determine whether improving QoL can prolong survival
Raffel et al. (84)	Multiple sclerosis	MSIS-29 physical and psychological score	Cox proportional hazards	N	Y	MSIS-29 only; not done for prEDSS	Y	Baseline MSIS-29 and prEDSS scores; 1-year worsening in MSIS-29-PHYS among patients with baseline scores of 85–100	Baseline MSIS-29 scores were prognostic of survival time, as was worsening of MSIS-29-PHYS in patients with baseline scores of 85–100. PROs can help detect changes in physical or psychological symptoms, support treatment decision-making, and serve as meaningful clinical outcomes due to their association with hard clinical endpoints
Kielbergerová et al. (50)	First-ever stroke	SF-36 and HADS	Cox proportional hazards	N	Y	SF-36; N/A for HADS	Y	SF-36	The presence of anxiety and depression, as measured by the HADS, was significantly associated with impaired QoL. Furthermore, impaired QoL, assessed using the SF-36, was an independent surrogate of mortality risk in stable patients at least six months after their first-ever stroke
Naess and Nyland (49)	First-ever stroke	SF-36 and NHP-I	Cox proportional hazards	N	Y		Y	NHP-I and SF-36 physical function and general health	Reduced HRQoL, as measured by either the SF-36 or NHP-I, was associated with long-term mortality in young adults with first-ever stroke. However, the results differed depending on which instrument was used, suggesting that it may be prudent to assess disease impact using more than one scale

15D, 15-dimensional instrument, ADL, Activities of Daily Living, ALSFRS-R, Revised Amyotrophic Lateral Sclerosis Functional Rating Scale, ALS-FTD-Q, Amyotrophic Lateral Sclerosis—Frontotemporal Dementia Questionnaire, AQoL, Assessment of Quality of Life, BDI, Beck Depression Index, CES-D, Center for Epidemiologic Studies Depression Scale, EORTC QLQ-BN20, European Organization for Research and Treatment of Cancer Brain Cancer Module, EORTC QLQ-C30, European Organization for Research and Treatment of Cancer Quality of Life Questionnaire, EQ-5D, EuroQoL five dimensions questionnaire, EQ-VAS, EuroQol Visual Analogue Scale, ESS, Epworth Sleepiness Scale, FACIT-F, Functional Assessment of Chronic Illness Therapy—Fatigue, FACT-Br, Functional Assessment of Cancer Therapy—Brain, FACT-G, Functional Assessment of Cancer Therapy—General, FrSBe, Frontal Systems Behavior Scale, FSS, Fatigue Severity Scale, HADS, Hospital Anxiety and Depression Scale, HUI, Health Utilities Index, IQCODE, Informant Questionnaire on Cognitive Decline in the Elderly, LASA, Linear Analog Scale Assessment, MDASI-BT, MD Anderson Symptom Inventory—Brain Tumor, MiND-B, Motor Neuron Disease Behavioral Instrument, MOS, Medical Outcomes Study, MQOL-SIS, McGill Quality of Life Questionnaire Single-Item Scale, MSIS, Multiple Sclerosis Impact Scale, PHQ, Patient Health Questionnaire, POMS-SF, Short Form of the Profile of Mood States, SDS, Symptom Distress Scale, SF-36, 36-Item Short-Form Survey, SRH, self-rated health, UPDRS, Unified Parkinson’s Disease Rating Scale, VAS, Visual Analogue Scale, NHP-I Nottingham Health Profile. Y = PROM statistically significant; N = PROM not statistically significant; N/A = not tested; ^a^List of PROMs assessed for their ability to predict mortality in the study. ^b^Specific instruments or components of PROMs that were statistically significant predictors of mortality, when not all PROMs evaluated in the study demonstrated predictive value.

### 3.4 Prediction of mortality

A total of 38 studies conducted univariable analyses, 43 conducted multivariable analyses, and 34 conducted both (Tables 1 and 2). Significant associations between PROMs and mortality were reported in 34 of 38 studies (89.5%) in univariable models and in 33 of 43 studies (76.7%) using multivariable models. Among the 34 studies that performed both univariable and multivariable analyses, 5 (14.7%) found significant associations in univariable analysis that were no longer significant after multivariable adjustment (25–29). One study reported significant variation by model type in multivariable analysis (30). In contrast, others reported variation by the presence of a lacunar infarct (31), depression (32), and cognitive impairment level (33). Age also influenced the magnitude of effect in at least one study (34).

In univariable analyses, 18 of 21 studies (85.7%) evaluating generic instruments and 18 of 20 (90.0%) evaluating condition-specific instruments reported significant associations with mortality (Table 1). These patterns were slightly attenuated in multivariable models: 19 of 27 studies (70.4%) evaluating generic instruments and 16 of 20 (80.0%) evaluating condition-specific instruments reporting significant associations (Table 1). PROMs independently predicted mortality in 11 of 13 studies on nervous system cancer, 10 of 14 on stroke, 6 of 7 on motor neuron disease, 4 of 5 on dementia, 1 of 3 on Parkinson’s disease, and 1 study on multiple sclerosis. Among the dementia studies that reported significant findings, two used proxy-reported PROMs (32, 35), one showed variation by the level of cognitive impairment (33), and another allowed either patient or proxy respondents (24). Notably, one study that did not find significant associations relied solely on patient-reported data (36). The instruments that most frequently predicted mortality in multivariable analyses were the EORTC QLQ for nervous system cancer (*n =* 7), the SF-36 for stroke (*n =* 6), and the ALSFRS-R for motor neuron disease (*n =* 2). Of seven studies that evaluated the associations between changes in PROMs over time and mortality, six reported significant associations in populations with nervous system cancer (*n =* 3), motor neuron disease (*n =* 2), and multiple sclerosis (*n =* 1).

Fifteen studies demonstrated inconsistent patterns in PROM’s associations with mortality, with only select instruments or subscores showing independent predictive value (Table 3 and Supplementary Table 4). After categorizing the PROM constructs for studies with mixed findings, 75.0% (9/12) of examined physical health measures or subscores were significantly associated with mortality, compared with 23.1% (3/13) of the emotional health measures and 66.7% (8/12) of other mental health–related subscores.

### 3.5 Quality assessment

**Figure 2:**
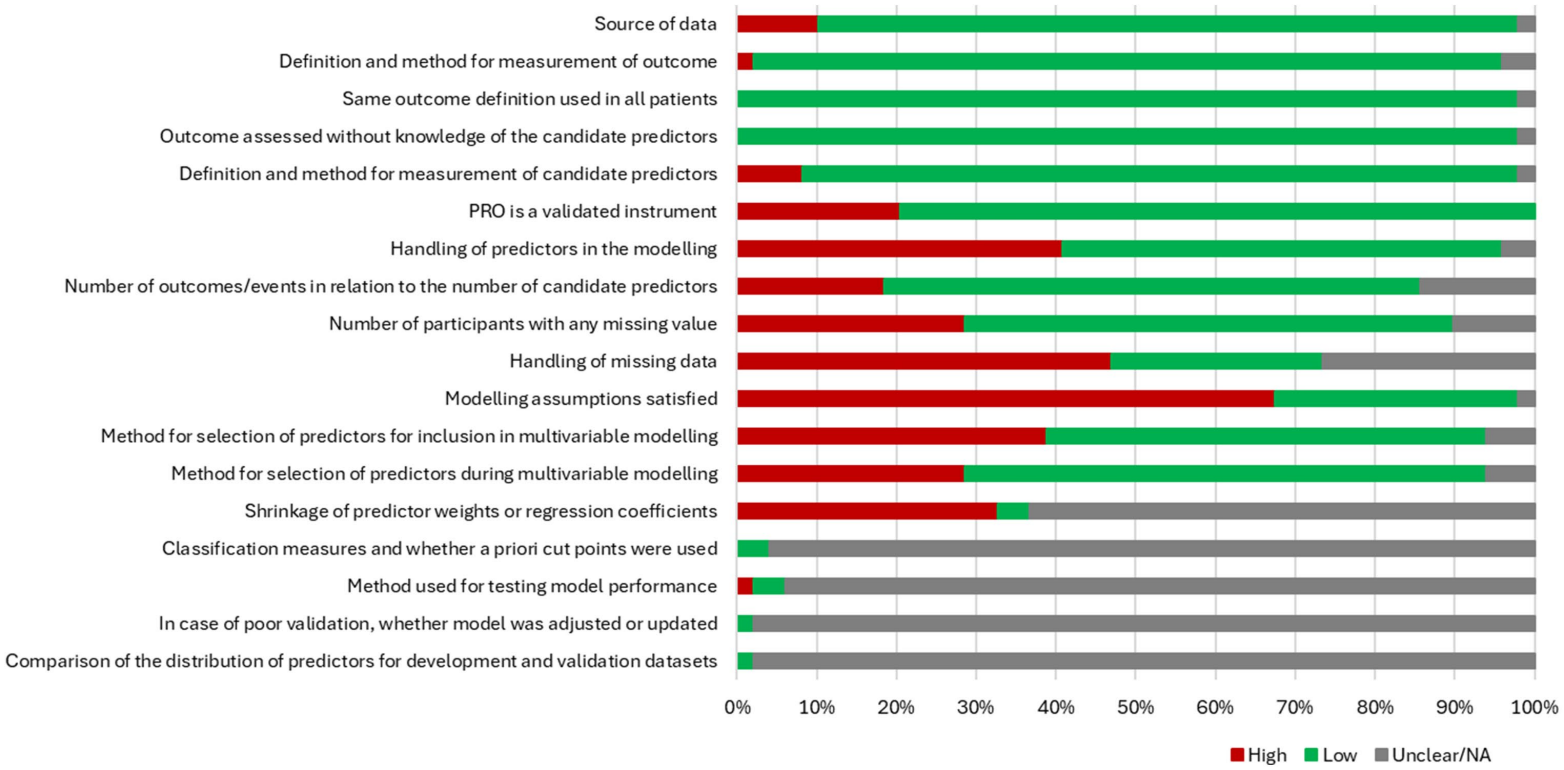
Risk-of-bias assessment using a modified CHARMS checklist. CHARMS: Critical Appraisal and Data Extraction for Systematic Reviews of Prediction Modeling Studies.

The quality and risk of bias were assessed using a modified CHARMS checklist (Figure 2). The majority of studies had low risk of bias based on source of data (*n =* 43, 87.8%), definition and method of measurement of survival/mortality (*n =* 46, 93.9%), and the validity of the PROM used (*n =* 39, 79.6%). Twenty studies (40.8%) had a high risk of bias in how they handled predictors in the model, with many studies categorizing PROMs without using established cut-points (i.e., median splits). Although 14 (28.6%) studies failed to report missing data, 23 (46.9%) demonstrated a high risk of bias in handling missing data in their models, with many using complete-case analysis without discussing how missing data could affect estimates and interpretation. Model assumptions were not reported in 33 (67.4%) studies; most studies had a low risk of bias in both the selection of predictors for multivariable models (*n =* 27, 55.1%) and the selection of final predictors in the models (*n =* 32, 65.3%). Finally, as most models were not prediction models, the questions related to assessing the quality of model performance and validation were not applicable.

## 4 Discussion

This systematic review is the first to comprehensively evaluate the prognostic value of PROMs in predicting mortality across various neurological conditions, mapping the current evidence on their use as predictors of survival in these patient populations. The review was originally designed to assess prediction models incorporating PROMs for potential integration into EHR systems to identify patients with neurological conditions at increased risk of mortality. However, only five studies aimed to develop a prediction model for clinical prognostication (37–41). Of these, two involved patients with nervous system cancer. They found only modest improvement in discrimination metrics (i.e., the C-index) when PROMs were added to established clinical prognostic factors, suggesting limited incremental value in some contexts (40–44). The others found that PROMs provided significant predictive information and supported their use in future clinical applications. None of the models were implemented clinically or into the EHR, and none provided information on challenges or opportunities for implementation, leaving many of our initial questions unanswered.

Rather than constructing prediction models, the majority of studies in this systematic review sought to identify independent associations between PROMs and mortality. Among studies that performed multivariable analyses, approximately three-quarters found that PROMs were independently associated with mortality. The strongest evidence for prognostic utility emerged in nervous system cancers, where condition-specific instruments, such as the EORTC QLQ, consistently and independently predicted mortality. Similarly, most studies on motor neuron disease demonstrated strong independent prognostic value, typically using condition-specific PROMs such as ALSFRS-R, with one study suggesting that its effect on overall survival may be mediated by faster progression through disease stages, each associated with increasing mortality risk (45). It is worth noting that most studies used clinician-reported ALSFRS-R; therefore, this systematic review included only a limited number of studies that employed the patient-reported version of ALSFRS-R, with two studies using the PRO-ACT database that included a mix of patient- and clinician-entered ALSFRS-R assessments (37, 45).

In contrast, findings were more variable in stroke populations, particularly by instrument type. Six studies evaluating the SF-36 demonstrated significant associations; three studies focused solely on physical function and symptoms (46–48), while two studies reported significance for the overall SF-36 scores and physical function but not for emotional health assessed by the SF-36 or HADS (49, 50). Other PROMs that incorporated physical components, such as self-perceived fatigue and AQoL, also demonstrated prognostic value (51, 52). Conversely, instruments lacking physical components, such as SRH, CES-D, and the self-reported acute psychological stress questionnaire, were consistently non-significant (28, 46, 53–56). Interestingly, age appeared to modify these associations: Ayerbe et al. (34) found depression (measured by HADS) to be significantly associated with mortality in stroke patients younger than 65 years, but not in older individuals. Most stroke study participants were older than those in nervous system cancer or ALS studies, except for one study limited to individuals under 50 (49). Prior research has suggested that older individuals may evaluate their health differently, often providing more favorable self-ratings that correlate less strongly with clinical measures and exhibit reduced predictive validity for mortality with advancing age (56–59). Consequently, the mixed associations observed in stroke may reflect a combination of impaired insight, altered health perceptions, and the overwhelming influence of acute cerebrovascular events on prognosis (28). It is also worth noting that our review identified a lack of condition-specific PROMs for cerebrovascular conditions, as none of the included studies utilized stroke-specific PROMs to predict mortality (52, 60). Disease-specific instruments with greater emphasis on physical function and symptom burden may enhance prognostic utility in this population.

Choice of PROMs may also be critical for mortality prediction in neurological diseases. Condition-specific instruments demonstrated stronger associations, with 80.0% of relevant studies reporting significant associations with mortality, compared with 70.4% of studies evaluating generic instruments. Condition-specific instruments are more likely to focus on physical symptoms and functioning than generic instruments. Of the studies evaluating separate dimensions, physical components were more likely to be associated with mortality than emotional health components, including depression, anxiety, and stress. Prior studies have likewise shown that self-rated physical health is more predictive of mortality than mental health, with a stronger association observed in men than in women (61, 62). Furthermore, one study found that despite moderate to high correlations between similar subscales of two HRQoL instruments, their prognostic value differed, suggesting that it may be prudent to assess disease impact using more than one scale when the primary goal of PROM collection is mortality prediction (49).

Finally, cognitive impairment may also impact the prognostic value of PROMs. Individuals with greater cognitive impairment were more likely to rate their health positively (36), and proxy-reported PROs, but not patient-reported PROs, predicted mortality in patients with dementia (35). Similarly, Walker et al. (33) observed that the predictive value of PROs declined with greater cognitive impairment, potentially reflecting reduced capacity to integrate less immediate factors into their overall health assessment (33). These findings suggest that future studies may benefit from incorporating cognitive function into evaluations of the prognostic value of PROs and from considering proxy reporting when appropriate. In addition, as noted in the risk-of-bias assessment, missing data posed a significant concern in interpreting study results. Missing data due to PROMs were reported to be more common among patients with cognitive impairment in a study evaluating the prognostic value of PROs in brain cancers (39). This pattern may partly be attributable to the mode of PROM delivery, which was observed to affect data completeness, with disease burden and physical and cognitive capacity suggested as potential contributing factors (63). Given the demonstrated predictive value of proxy-reported PROs in populations with dementia and cognitive impairment, broader use of proxy reporting, along with careful consideration of PROM delivery methods, could enhance data completeness and reliability.

Several limitations of our review should be considered. First, only studies published after 2002 were included, as the use of PROs in healthcare was relatively uncommon before the Institute of Medicine highlighted patient-centeredness as one of the six aims for quality of healthcare (21). Although earlier literature demonstrated significant associations between PROs, particularly SRH, and mortality across different populations (64–66), these studies did not evaluate prediction models for use in large healthcare settings. Second, limiting inclusion to full-text articles may have introduced selection bias; however, this was necessary given the impracticality of screening more than 55,000 records otherwise. Nevertheless, a thorough review of references was conducted, and 16 additional manuscripts not captured during initial screening were included.

The heterogeneity in study design, PROM instruments, timing of administrations, and statistical methods precluded quantitative synthesis in a meta-analysis. This heterogeneity likely reflects differences in disease mechanisms and clinical trajectories across neurological conditions, as well as variations in the locations and eras in which studies were conducted, during which the selection, availability, and popularity of PROM instruments may have differed. Consequently, the evidence was synthesized using a descriptive approach stratified primarily by neurological condition, highlighting an important opportunity to improve comparability across future studies by standardizing PROM selection and administration within and across neurological conditions.

Several limitations of the included studies were also identified through the CHARMS-based risk-of-bias assessment. The majority of studies did not explicitly report evaluation of key modeling assumptions, such as proportional hazards, and were therefore conservatively rated as not meeting these criteria. Similarly, handling of missing data was frequently insufficiently reported, with many studies relying on complete-case analysis or providing no description of how missing data were handled. Substantial variation was also observed in predictor handling, including whether PROMs were modeled as continuous variables or categorized using established, validated, or otherwise justified cut-points, as well as in the transparency of predictor selection for inclusion in multivariable models. However, most included studies were not designed as formal prediction model studies but rather aimed to examine prognostic associations between PROMs and mortality; therefore, comprehensive reporting of model assumptions, performance metrics, and validations may have been outside their stated objectives. Nevertheless, future studies seeking to translate PROMs into clinically useful prognostic tools would benefit from greater adherence to established reporting guidelines to improve transparency and interpretability, thereby strengthening the overall evidence base.

No studies evaluated the association between PROs and mortality in four of the included neurological conditions (i.e., headache, diabetic neuropathy, Guillain–Barré syndrome, and epilepsy), limiting the generalizability across the full spectrum of neurological diseases and highlighting an important gap for future research on the prognostic value of PROMs. Finally, no studies evaluated predictive models implemented in healthcare settings, which was one of the original aims of this systematic review. Further research is needed to elucidate challenges and opportunities for integrating these emerging models into clinical practice. Despite these limitations, this review provides a comprehensive descriptive overview of PROMs used to predict mortality across a wide range of neurological conditions.

## 5 Conclusion

Overall, this systematic review found that PROMs independently predict mortality, particularly in nervous system cancers and motor neuron disease. In contrast, findings were more variable in stroke and dementia depending on the PROM instruments administered, where factors such as cognitive impairment, altered health perceptions, lack of condition-specific instruments, and age may influence prognostic performance. These results underscore the importance of selecting appropriate PROMs, accounting for relevant patient characteristics, and incorporating proxy reporting when necessary. Furthermore, no eligible studies evaluated the prognostic value of PROMs for mortality in epilepsy, headache, Guillain–Barré syndrome, or diabetic neuropathy, highlighting important gaps in the current evidence base. PROMs may serve as valuable tools for screening and monitoring patients’ symptoms and as indicators of mortality risk. As the use of PROMs in neurological care continues to expand, future studies should extend the evaluation of their prognostic value for mortality to currently underrepresented neurological conditions and rigorously assess their added prognostic value beyond traditional clinical predictors.

## Data Availability

The original contributions presented in the study are included in the article/Supplementary material, further inquiries can be directed to the corresponding author.
