# Supplementary material for: Can patient-reported outcome measures predict mortality in neurological populations? A systematic review
Source: Front Neurol. 2026 Jan 28;17:1705393. doi: 10.3389/fneur.2026.1705393 (PMC12890656; doi:10.3389/fneur.2026.1705393)
Supplement: Supplementary file 1 [file Table_1.DOCX]

**Supplemental Table 1. Extraction Sheet Variables**

| **Topic** | **Field** |
| --- | --- |
| Study Details | Sponsorship source |
|  | Country |
|  | Setting |
|  | Comments |
|  | Author name |
|  | Author institution |
|  | Publication year |
| Methods | Design |
|  | Recruitment method (e.g. consecutive participants, location, number of centers) |
|  | Study aim(s) |
|  | Outcome definition (e.g. 3-month mortality, single or combined endpoint) |
|  | PRO definition (e.g. PRO dichotomized for prediction model, or PRO continuous) |
|  | Modelling method (e.g. logistic, survival, machine learning, etc) |
| Population | Participant clinical condition description |
|  | Number of participants |
|  | Study dates |
|  | Number and percentage deceased |
|  | Baseline characteristics (PRO, mean age, percent female, condition-specific severity) |
| Patient-Reported Outcome | How PROs were completed (e.g. in-person, electronically, etc) |
|  | Timing of PROs (e.g., at patient presentation, at diagnosis, at treatment initiation) |
|  | Number of participants with completed PROs |
|  | Other variables included in prediction model |
|  | Number of participants with any missing value (predictor or outcome) |
|  | Interpretation of presented models (confirmatory, i.e., model useful for practice vs exploratory, more research needed) |
|  | Clinical interpretation (ie how will these PRO results be used clinically) |
| Results | Effect estimate, 95% confidence interval, and p-value for relationship between PRO and mortality |
|  | c-statistic, if available |

**Supplemental Table 2. Domains for risk of bias assessment**

|  |  | **Domain** | **HIGH Quality Description** | **LOW Quality Description** | **Comments** |
| --- | --- | --- | --- | --- | --- |
| Source of data | 1 | Source of data (e.g., cohort, case-control, randomized trial participants, or registry data) | prospective cohort, nested case-control, case-cohort, RCT | non-nested case-control, registry, retrospective cohort |  |
| Outcome(s) to be predicted | 2 | Definition and method for measurement of outcome | Clearly stated the definition of mortality; in the definition of disease-free survival, clearly stated which events were included. | The definition of mortality was NOT clear; in the definition of disease-free survival, DID NOT clearly state which events were included. | Definition and measurement of the outcome event should correspond to the outcome definition of the systematic review question. Note, we will include any definition of mortality (all-cause mortality, disease-free survival etc.) |
|  | 3 | Was the same outcome definition (and method for measurement) used in all patients? | Yes. | No. |  |
|  | 4 | Was the outcome assessed without knowledge of the candidate predictors (i.e., blinded)? | Yes. The assessor of the outcome occurrence was blinded to the ascertainment of the predictor. | No. The assessor of the outcome occurrence was NOT blinded to the ascertainment of the predictor. | For mortality, blinded outcome assessment is less important. However, cause-specific mortality may include subjective interpretation so that knowledge of predictors could bias outcome assignment. |
| Candidate predictors (or index tests) | 5 | Definition and method for measurement of candidate predictors | Clearly stated the name, version, administration mode of PROs | DID NOT provide information on name, version, administration mode of PROs | Different definitions and measurement methods of candidate predictors are a potential source of heterogeneity and thus risk of bias, and the use of different measurement methods may affect the strength of predictors and influence whether the predictors ultimately are included in the prediction model. |
|  | 6 | PRO is a validated instrument | Yes, and references were provided to demonstrate validity/reliability | No |  |
|  | 7 | Handling of predictors in the modelling (e.g., continuous, linear, non-linear transformations or categorized) | Continuous variables are NOT categorized, or categorization is based on established cut-points (with references) | Continuous variables are categorized; or categorization is based on cut-points from the data or one study that used cut-points from their data. | Or call it LOW quality only if the categorization is based on cut points based on data from one study |
| Sample size | 8 | Number of outcomes/events in relation to the number of candidate predictors (Events Per Variable) | Events Per Variable >=10 or adequate sample size for prediction models based on QHS sample size calculator (if able, <https://www.lerner.ccf.org/quantitative-health/software/#research-calculators>) | Events Per Variable <10 or inadequate sample size | The number of variables includes all candidate predictors, transformations for continuous predictors, indicator variables for categorical predictors, and interactions examined. |
| Missing data | 9 | Number of participants with any missing value (include predictors and outcomes) | Reported number of participants with any missing value (include predictors and outcomes) and detail on whether missing data may reasonably be missing at random (by comparison of the participants with and without missing values). | NOT reported number of participants with any missing value (include predictors and outcomes) and detail on whether missing data may reasonably be missing at random. |  |
|  | 10 | Handling of missing data (e.g., complete-case analysis, imputation, or other methods) | Missing data handled by multiple imputation or other preferred methods. | Complete-case analysis (participants with a missing value were deleted). | Where missing data >5% |
| Model development | 11 | Modelling assumptions satisfied | Yes. | No/Not reported. |  |
|  | 12 | Method for selection of predictors for inclusion in multivariable modelling (e.g., all candidate predictors, pre-selection based on unadjusted association with the outcome) | Used all candidate predictors; or justified inclusion of predictors based on clinical significance | Screening or pre-selection based on univariable significance. | The risk of predictor selection bias is greater in smaller datasets and when there are notably weak predictors. |
|  | 13 | Method for selection of predictors during multivariable modelling (e.g., full model approach, backward or forward selection) | Full model approach (pre-specifies all predictors in the final model and no predictors are omitted, which avoids predictor selection bias) | Backward or forward selection. | There is no consensus on the best method. Overfitting may arise with backward or forward selection, particularly in small datasets. |
|  | 14 | Shrinkage of predictor weights or regression coefficients (e.g., no shrinkage, uniform shrinkage, penalized estimation) | Shrinkage techniques were used to address possible overfitting of a model. | Shrinkage techniques were NOT used. | The need for use of shrinkage methods increases with smaller datasets. |
| Model performance | 15 | Classification measures (e.g., sensitivity, specificity, predictive values, net reclassification improvement) and whether a priori cut points were used | Reported classification measures (e.g., sensitivity, specificity, predictive values, net reclassification improvement); a priori cut points were used. | NOT reported classification measures (e.g., sensitivity, specificity, predictive values, net reclassification improvement); cut points were chosen from data which can produce over-optimistic and biased performance. |  |
| Model evaluation | 16 | Method used for testing model performance: development dataset only (random split of data, resampling methods, e.g., bootstrap or cross-validation, none) or separate external validation (e.g., temporal, geographical, different setting, different investigators) | Internal validation using bootstrap or cross-validation; or External validation with an independent dataset | Model evaluation with the development dataset (internal validation), or single split; No external validation; or no model validation. |  |
|  | 17 | In case of poor validation, whether model was adjusted or updated (e.g., intercept recalibrated, predictor effects adjusted, or new predictors added) | Model was adjusted or updated in case of poor validation. | Model was NOT adjusted or updated in case of poor validation. | Not applicable to studies without model validation or validation performs well. |
| Results | 18 | Comparison of the distribution of predictors (including missing data) for development and validation datasets | Reported differences in frequency (binary) and distribution (continuous) of the predictors and outcomes across the study samples. | NOT reported differences in frequency (binary) and distribution (continuous) of the predictors and outcomes across the study samples. | Not applicable to studies without model validation. |

**Supplementary Table 3. Classifications of PROM Instruments**

| **PROM Instrument** | **Classification (Generic/Condition-Specific)** | **Target Domain(s)** | **Reference(s)** |
| --- | --- | --- | --- |
| SRH | Generic | General health perceptions | Hillen et al., 2003[1]  Walker et al., 2004[2]  Xie et al., 2008[3]  Nielsen et al., 2016[4]  Mavaddat et al., 2016[5]  Araujo et al., 2019 [6] |
| SRH Transition | Generic | Change in perceived health | Hillen et al., 2003[1] |
| EQ-5D | Generic | HRQOL | González-Vélez et al., 2015[7] |
| EQ-VAS | Generic | HRQOL | Phung et al., 2018[8] |
| SF-36 | Generic | HRQOL | Naess and Nyland, 2013[9]  Del Aguila et al., 2003[10]  Kielbergerová et al., 2015[11] |
| SF-36 (excluding role-physical) | Generic | HRQOL | Mead et al., 2011[12] |
| SF-36 mental component summary | Generic | Mental health | Grool et al., 2012[13]  Bosma et al., 2009[14] |
| SF-36 physical functioning | Generic | Physical function | Bell et al., 2013[15]  Hubbard et al., 2016[16] |
| 15D | Generic | HRQOL | Liira et al., 2018[17] |
| MQoL-SIS | Generic | HRQOL | Lou et al., 2010[18] |
| HUI | Generic | HRQOL | McCarter et al., 2006[19] |
| AQoL | Generic | HRQOL | Sturm et al., 2002[20] |
| NHP-I | Generic | HRQOL | Naess and Nyland, 2013[9] |
| LASA | Generic | General health perceptions | Brown et al., 2005[21] |
| PHQ-9 | Generic | Depression | Thakore and Pioro, 2016[22] |
| HADS | Generic | Anxiety, depression | Ayerbe et al., 2014[23]  Kielbergerová et al., 2015[11] |
| CES-D | Generic | Depression | Bell et al., 2013[15]  Ertel et al., 2007[24]  Winovich et al., 2017[25] |
| BDI-II | Generic | Depression | Noll et al., 2019[26]  John et al., 2021[27] |
| POMS-SF depression | Generic | Depression | Brown et al., 2005[21] |
| POMS-SF fatigue | Generic | Fatigue | Brown et al., 2005[21] |
| SDS-fatigue | Generic | Fatigue | Brown et al., 2005[21] |
| FSS | Generic | Fatigue | Wang et al., 2022[28] |
| Self-perceived fatigue | Generic | Fatigue | Glader et al., 2002[29] |
| MOS social support | Generic | Social support | Bell et al., 2013[15] |
| ESS | Generic | Sleepiness | Brown et al., 2005[21] |
| Self-reported acute psychological stress questionnaire | Generic | Psychological stress | Mokhber et al., 2021[30] |
| Dependency on primary ADL | Generic | Activities of daily living | Glader et al., 2002[29] |
| ALSFRS-R | Condition-Specific | Physical function in ALS | Ackrivo et al., 2019[31]  Thakore et al., 2018[32]  Thakore and Pioro, 2016[22]  Van Eijk et al., 2024[33] |
| ALS-FTD-Q | Condition-Specific | Behavioral impairment in ALS | Xu et al., 2017[34] |
| MiND-B | Condition-Specific | Behavioral impairment in ALS | Xu et al., 2017[34] |
| ALS severity score | Condition-Specific | Disease severity in ALS | Del Aguila et al., 2003[10] |
| VAS for cramps | Condition-Specific | Symptom rating in ALS | Paillisse et al., 2005[35] |
| VAS for stiffness | Condition-Specific | Symptom rating in ALS | Paillisse et al., 2005[35] |
| VAS for tiredness | Condition-Specific | Symptom rating in ALS | Paillisse et al., 2005[35] |
| VAS for fasciculations | Condition-Specific | Symptom rating in ALS | Paillisse et al., 2005[35] |
| FrSBe | Condition-Specific | Executive function/neurobehavioral | Chio et al., 2012[36] |
| EORTC-QLQ | Condition-Specific | HRQOL in cancer | Paquette et al., 2016[37]  Mauer et al., 2007[38]  Mauer et al., 2008[39]  Armstrong et al., 2013[40]  Ediebah et al., 2015[41]  Quinten et al., 2014[42]  Wefel et a., 2021[43] |
| FACT-Br | Condition-Specific | HRQOL in brain cancer | Peters et al., 2014[44]  Brown et al., 2005[21] |
| FACT-G | Condition-Specific | HRQOL in cancer | Peters et al., 2014[44]  Sehlen et al., 2003[45] |
| FACIT-F | Condition-Specific | Fatigue in cancer | Peters et al., 2014[44] |
| MDASI-BT | Condition-Specific | Symptom burden in brain tumor | Wefel et al., 2021[43] |
| Stavanger Sleepiness Questionnaire REM sleep behavior disorder subscore | Condition-Specific | Sleep Behavior in Parkinson's Disease | Forsaa et al., 2010[46] |
| UPDRS I psychosis subscore | Condition-Specific | Psychotic symptoms in Parkinson's Disease | Forsaa et al., 2010[46] |
| UPDRS II | Condition-Specific | Activities of daily living in Parkinson's Disease | Wang et al., 2022[28]  Krishnan et al., 2019[47] |
| MSIS-29 physical score | Condition-Specific | Physical function in Multiple sclerosis | Raffel et al., 2017[48] |
| MSIS-29 psychological score | Condition-Specific | Psychological function in Multiple sclerosis | Raffel et al., 2017[48] |
| IQCODE | Condition-Specific | Cognitive function | Zietemann et al., 2018[49] |

*15D* 15-dimensional Instruemnt*, ADL* Activities of Daily Living, *ALSFRS-R* Revised Amyotrophic Lateral Sclerosis Functional Rating Scale, *ALS-FTD-Q* Amyotrophic Lateral Sclerosis – Frontotemporal Dementia Questionnaire, *AQoL* Assessment of Quality of Life, *BDI* Beck Depression Index, *CES-D* Center for Epidemiologic Studies Depression Scale, *EORTC QLQ-BN20* European Organization for Research and Treatment of Cancer Brain Cancer Module, *EORTC QLQ-C30* European Organization for Research and Treatment of Cancer Quality of Life questionnaire, *EQ-5D* EuroQoL five dimensions questionnaire, *EQ-VAS* EuroQol Visual Analogue Scale, *ESS* Epworth Sleepiness Scale, *FACIT-F* Functional Assessment of Chronic Illness Therapy – Fatigue, *FACT-Br* Functional Assessment of Cancer Therapy – Brain, *FACT-G* Functional Assessment of Cancer Therapy – General, *FrSBe* Frontal Systems Behavior Scale, *FSS* Fatigue Severity Scale, *HADS* Hospital Anxiety and Depression Scale, *HUI* Health Utilities Index, *IQCODE* Informant Questionnaire on Cognitive Decline in the Elderly, *LASA* Linear Analog Scale Assessment, *MDASI-BT* MD Anderson Symptom Inventory – Brain Tumor, *MiND-B* Motor Neuron Disease Behavioural instrument, *MOS* Medical Outcomes Study, *MQOL-SIS* McGill Quality of Life Questionnaire Single-Item Scale, *MSIS* Multiple Sclerosis Impact Scale, *PHQ* Patient Health Questionnaire, *POMS-SF* Short Form of the Profile of Mood States, *SDS* Symptom Distress Scale, *SF-36* 36-Item Short-Form Survey, *SRH* Self-rated health, *UPDRS* Unified Parkinson's Disease Rating Scale, *VAS* Visual Analogue Scale, NHP-I Nottingham Health Profile.

**Supplementary Table 4. Breakdown of Significant PROM Associations with Mortality Among Studies Reporting Mixed Findings**

|  |  | Physical health/symptoms | | Emotional health  (Depression, anxiety, stress, etc) | | Other mental health  (Executive function, neurobehavioral, etc) | |
| --- | --- | --- | --- | --- | --- | --- | --- |
| Study | All Instrument(s) examined in the study | Examined?* | Independent predictor of mortality?** | Examined?* | Independent predictor of mortality?** | Examined?* | Independent predictor of mortality?** |
| Armstrong et al., 2013[40] | EORTC QLQ-C30/BN20 | Y | EORTC physical function, nausea/vomiting item, early changes in motor dysfunction, and hairloss item | Y | N | Y | EORTC early changes in cognitive functioning scale |
| Bell et al., 2013[15] | SF-36 physical functioning, CES-D, MOS social support | Y | SF-36 physical function | Y | N | Y | N |
| DelAguila et al., 2003[10] | SF-36 and ALS severity score | Y | ALS severity score, SF-36 physical health summary | Y | N | N | N/A |
| Forsaa et al., 2010[46] | Stavanger Sleepiness Questionnaire REM sleep behavior disorder subscore and UPDRS I psychosis subscore | N | N/A | N | N/A | Y | UPDRS I psychosis subscore |
| Mauer et al., 2007[38] | EORTC QLQ-C30/BN20 | Y | N | Y | N | Y | EORTC cognitive functioning, social functioning |
| Mauer et al., 2007[39] | EORTC QLQ-C30/BN20 | Y | EORTC weakness of legs | Y | EORTC emotional functioning, future uncertainty | Y | EORTC cognitive functioning |
| Mead et al., 2011[12] | SF-36 (excluding role-physical) | Y | SF-36 vitality | Y | N | Y | SF-36 social role |
| Paquette et al., 2016[37] | EORTC QLQ-C30/BN20 | Y | N | Y | EORTC future uncertainty | Y | N |
| Peters et al., 2014[44] | FACT-G, FACT-Br and FACIT-F | Y | FACIT-fatigue | Y | N | Y | N |
| Quinten et al., 2014[42] | EORTC QLQ-C30 | Y | N | Y | N | Y | EORTC cognitive functioning |
| Xu et al., 2017[34] | ALS-FTD-Q and MiND-B | N | N | N | N | Y | ALS-FTD-Q |
| Wefel et al., 2021 [43] | EORTC QLQ-C30/BN20 and MDASI-BT | Y | EORTC physical function | Y | N | Y | Early changes in EORTC communication deficit and MDASI-BT cognitive factor |
| Kielbergerová et al., 2015[11] | SF-36 and HADS | N | N/A | Y | N | N | N/A |
| Naess and Nyland, 2013[9]*** | SF-36 | Y | SF-36 physical function | Y | N | Y | N |
|  | NHP-I | Y | NHP-I pain, sleep disturbances | Y | NHP-I social isolations | N | N/A |
| **Total** | 15 | 12 | 9 | 13 | 3 | 12 | 8 |

* "Y" indicates the domain was examined in the study; "N" indicates it was not examined. ** If the domain was significantly associated with mortality, the corresponding PROM or component is listed; "N" indicates the domain was examined but not significant. ***SF-36 and NHP-I were examined in separate model.

**Supplemental Appendix 1. Search Queries**

Ovid MEDLINE(R) ALL <1946 to November 08, 2024> (498 results)

1 Patient Outcome Assessment/ 6246

2 Patient Reported Outcome Measures/ 16668

3 ((patient reported or patient driven or proxy reported or self reported) adj5 (outcome* or measure*)).mp. 74484

4 exp "Quality of Life"/ 296536

5 "Quality of Life".mp. 503478

6 Visual Analog Scale/ 4182

7 (VAS or "Visual Analog Scale").mp. 94633

8 ("Activities of daily living" or ADL* or AIM-D or ALSFRS-R or BAI or BDI or "Brief Fatigue Inventory" or BFI or CES or "Cornell-Brown Scale for Quality of Life" or CFQ or QOL-AD or "Diabetes Distress Scale" or DDS or DEMQOL or "Diabetes Symptom Self-Care Inventory" or DSSCI or EDDS or EMIQ or EORTC or ESAS or EQ5D* or EQ-5D* or EuroQOL or FACIT or FACT or FAMS or FIS or FSMC or FSS or GAD2 or GAD-2 or GAD7 or GAD-7 or GNDS or HADS or HALEMS or HAQUAMS or HIT-6 or MDASI or "MD Anderson Symptom Inventory" or "Medical Outcomes Study" or MFIQ or MFIS or MIDAS or "Migraine-specific quality of life" or MSQ or MOCA or MOS or MPFID or MS-HRS or MSNQ or MSIS* or MSWS* or MSQLI or MSQOL or MusiQOL or NDDI or Neuro-QoL or NMSQ or "Numerical Rating Scale" or NRS or "Patient Global Impression of Change" or PGIC or PGI-C or PDDS or PHQ2 or PHQ-2 or PHQ9 or PHQ-9 or "Problem Areas in Diabetes" or PAID or PDQ* or PROMIS or PRO-PD or PRIMUS or QOLAS or QOL-D or QOLIE or SA-SIP30 or SATIS-Stroke or "Short Form Health Survey" or SF-36 or SF-12 or SIS* or "Spitzer Quality of Life Index" or SQLI or SS-QOL or STAI or STA-I or "Symptom Checklist 92" or SCL-92 or UKNDS or UNDS or UPDRS or VR-12 or Veteran-12 or VR12 or Veteran12 or WEIMuS or WHODAS or "WHO-5 Well-Being Index" or WHOQOL or WPAI).mp. 748796

9 or/1-8 1280670

10 exp Motor Neuron Disease/ 36105

11 (Motor neuron disease* or amyotrophic lateral sclerosis or primary lateral sclerosis or progressive bulbar palsy or progressive muscular atrophy).kw,ti. 21542

12 exp Diabetic Neuropathies/ 28349

13 (asymmetric diabetic proximal motor neuropath* or diabetic amyotroph* or diabetic asymmetric polyneuropath* or diabetic autonomic neuropath* or diabetic mononeuropath* or diabetic neuralg* or diabetic neuropath* or diabetic polyneuropath* or symmetric diabetic proximal motor neuropath*).kw,ti. 6645

14 exp Nervous System Neoplasms/ 262524

15 (Nervous System Cancer* or Brain tumor or brain neoplasm* or glioma* or meningioma* or peripheral nerve tumor* spinal cord tumor*).kw,ti. 79615

16 Alzheimer Disease/ 129041

17 (Alzheimer* disease or dementia or mild cognitive impairment).kw,ti. 184643

18 exp Guillain-Barre Syndrome/ 6659

19 (acute autoimmune neuropathy or acute inflammatory demyelinating or acute inflammatory polyneuropathy or acute inflammatory polyradiculoneuropathy or guillain*barre syndrome).kw,ti. 2445

20 exp Epilepsy/ 131057

21 (aura* or epileps* or seizure disorder*).kw,ti. 100115

22 exp Headache/ 32760

23 exp Headache Disorders/ 42097

24 (cephalalgia* or cephalgia* or cephalodynia* or cranial pain* or head pain* or headache* or hemicrania or migraine).kw,ti. 54951

25 exp Multiple Sclerosis/ 73767

26 (disseminated sclerosis or multiple sclerosis).kw,ti. 69999

27 exp Parkinson Disease/ 87971

28 (paralysis agitan* or parkinson* disease* or primary parkinsonism).kw,ti. 85804

29 exp Stroke/ 186173

30 (Stroke or ischemic stroke or intracerebral hemorrhage or subarachnoid hemorrhage or transient ischemic attack).kw,ti. 188465

31 or/10-30 1245718

32 exp Algorithms/ 483060

33 exp Risk Factors/ 1010284

34 exp "Sensitivity and Specificity"/ 669663

35 (algorithm* or "artificial intelligence" or "clinical decision support" or "machine learning" or informatics or model* or nomogram* or predict* or risk* or "roc curve" or "sensitivity and specificity").mp. 9771767

36 or/32-35 9846449

37 exp Survival Rate/ 193258

38 (mortality or survival*).mp. 2651822

39 37 or 38 2651822

40 exp Adult/ 8237648

41 (adult* or age* or elderly).mp. 14474787

42 40 or 41 14474790

43 9 and 31 and 36 and 39 and 42 3597

44 limit 43 to (full text and human and english language and yr="2002 -Current") 498

Embase <1974 to 2024 November 08> (4328 results)

1 exp outcome assessment/ 937001

2 patient-reported outcome/ 67178

3 ((patient reported or patient driven or proxy reported or self reported) adj5 (outcome* or measure*)).mp. 123251

4 exp "quality of life"/ 726284

5 "Quality of Life".mp. 886750

6 visual analog scale/ 139909

7 (VAS or "Visual Analog Scale").mp. 200151

8 ("Activities of daily living" or ADL* or AIM-D or ALSFRS-R or BAI or BDI or "Brief Fatigue Inventory" or BFI or CES or "Cornell-Brown Scale for Quality of Life" or CFQ or QOL-AD or "Diabetes Distress Scale" or DDS or DEMQOL or "Diabetes Symptom Self-Care Inventory" or DSSCI or EDDS or EMIQ or EORTC or ESAS or EQ5D* or EQ-5D* or EuroQOL or FACIT or FACT or FAMS or FIS or FSMC or FSS or GAD2 or GAD-2 or GAD7 or GAD-7 or GNDS or HADS or HALEMS or HAQUAMS or HIT-6 or MDASI or "MD Anderson Symptom Inventory" or "Medical Outcomes Study" or MFIQ or MFIS or MIDAS or "Migraine-specific quality of life" or MSQ or MOCA or MOS or MPFID or MS-HRS or MSNQ or MSIS* or MSWS* or MSQLI or MSQOL or MusiQOL or NDDI or Neuro-QoL or NMSQ or "Numerical Rating Scale" or NRS or "Patient Global Impression of Change" or PGIC or PGI-C or PDDS or PHQ2 or PHQ-2 or PHQ9 or PHQ-9 or "Problem Areas in Diabetes" or PAID or PDQ* or PROMIS or PRO-PD or PRIMUS or QOLAS or QOL-D or QOLIE or SA-SIP30 or SATIS-Stroke or "Short Form Health Survey" or SF-36 or SF-12 or SIS* or "Spitzer Quality of Life Index" or SQLI or SS-QOL or STAI or STA-I or "Symptom Checklist 92" or SCL-92 or UKNDS or UNDS or UPDRS or VR-12 or Veteran-12 or VR12 or Veteran12 or WEIMuS or WHODAS or "WHO-5 Well-Being Index" or WHOQOL or WPAI).mp. 972663

9 or/1-8 2713746

10 exp motor neuron disease/ 63653

11 (Motor neuron disease* or amyotrophic lateral sclerosis or primary lateral sclerosis or progressive bulbar palsy or progressive muscular atrophy).kw,ti. 28955

12 exp diabetic neuropathy/ 31207

13 (asymmetric diabetic proximal motor neuropath* or diabetic amyotroph* or diabetic asymmetric polyneuropath* or diabetic autonomic neuropath* or diabetic mononeuropath* or diabetic neuralg* or diabetic neuropath* or diabetic polyneuropath* or symmetric diabetic proximal motor neuropath*).kw,ti. 10124

14 exp nervous system tumor/ 575988

15 (Nervous System Cancer* or Brain tumor or brain neoplasm* or glioma* or meningioma* or peripheral nerve tumor* spinal cord tumor*).kw,ti. 103860

16 exp Alzheimer disease/ 270051

17 (Alzheimer* disease or dementia or mild cognitive impairment).kw,ti. 255400

18 exp Guillain Barre syndrome/ 19760

19 (acute autoimmune neuropathy or acute inflammatory demyelinating or acute inflammatory polyneuropathy or acute inflammatory polyradiculoneuropathy or guillain*barre syndrome).kw,ti. 4523

20 exp epilepsy/ 289290

21 (aura* or epileps* or seizure disorder*).kw,ti. 137100

22 exp "headache and facial pain"/ 406580

23 (cephalalgia* or cephalgia* or cephalodynia* or cranial pain* or head pain* or headache* or hemicrania).kw,ti. 44541

24 exp multiple sclerosis/ 168376

25 (disseminated sclerosis or multiple sclerosis).kw,ti. 106683

26 exp Parkinson disease/ 206850

27 (paralysis agitan* or parkinson* disease* or primary parkinsonism).kw,ti. 129580

28 exp cerebrovascular accident/ 355111

29 (Stroke or ischemic stroke or intracerebral hemorrhage or subarachnoid hemorrhage or transient ischemic attack).kw,ti. 291643

30 or/10-29 2445306

31 exp algorithm/ 677448

32 exp risk factor/ 1487943

33 exp "sensitivity and specificity"/ 530789

34 (algorithm* or "artificial intelligence" or "clinical decision support" or "machine learning" or informatics or model* or nomogram* or predict* or risk* or "roc curve" or "sensitivity and specificity").mp. 13299304

35 or/31-34 13364026

36 exp survival rate/ 328043

37 (mortality or survival*).mp. 3967148

38 36 or 37 3967148

39 exp adult/ 11624435

40 (adult* or age* or elderly).mp. 18053686

41 39 or 40 18053717

42 9 and 30 and 35 and 38 and 41 31929

43 limit 42 to (full text and human and english language and yr="2002 -Current") 4328

Cochrane Central Register of Controlled Trials Issue 10 of 12, October 2024 and Cochrane Database of Systematic Reviews Issue 11 of 12, November 2024 (1378 results)

ID Search Hits

#1 MeSH descriptor: [Patient Outcome Assessment] this term only 505

#2 MeSH descriptor: [Patient Reported Outcome Measures] this term only 1914

#3 ((patient reported or patient driven or proxy reported or self reported) NEAR5 (outcome* or measure*)) 463

#4 MeSH descriptor: [Quality of Life] explode all trees 44954

#5 "Quality of Life":ti,ab,kw 168137

#6 MeSH descriptor: [Visual Analog Scale] this term only 1361

#7 (VAS or "Visual Analog Scale"):ti,ab,kw 87832

#8 ("Activities of daily living" or ADL* or AIM-D or ALSFRS-R or BAI or BDI or "Brief Fatigue Inventory" or BFI or CES or "Cornell-Brown Scale for Quality of Life" or CFQ or QOL-AD or "Diabetes Distress Scale" or DDS or DEMQOL or "Diabetes Symptom Self-Care Inventory" or DSSCI or EDDS or EMIQ or EORTC or ESAS or EQ5D* or EQ-5D* or EuroQOL or FACIT or FACT or FAMS or FIS or FSMC or FSS or GAD2 or GAD-2 or GAD7 or GAD-7 or GNDS or HADS or HALEMS or HAQUAMS or HIT-6 or MDASI or "MD Anderson Symptom Inventory" or "Medical Outcomes Study" or MFIQ or MFIS or MIDAS or "Migraine-specific quality of life" or MSQ or MOCA or MOS or MPFID or MS-HRS or MSNQ or MSIS* or MSWS* or MSQLI or MSQOL or MusiQOL or NDDI or Neuro-QoL or NMSQ or "Numerical Rating Scale" or NRS or "Patient Global Impression of Change" or PGIC or PGI-C or PDDS or PHQ2 or PHQ-2 or PHQ9 or PHQ-9 or "Problem Areas in Diabetes" or PAID or PDQ* or PROMIS or PRO-PD or PRIMUS or QOLAS or QOL-D or QOLIE or SA-SIP30 or SATIS-Stroke or "Short Form Health Survey" or SF-36 or SF-12 or SIS* or "Spitzer Quality of Life Index" or SQLI or SS-QOL or STAI or STA-I or "Symptom Checklist 92" or SCL-92 or UKNDS or UNDS or UPDRS or VR-12 or Veteran-12 or VR12 or Veteran12 or WEIMuS or WHODAS or "WHO-5 Well-Being Index" or WHOQOL or WPAI):ti,ab,kw 119468

#9 {OR #1-#8} 310524

#10 MeSH descriptor: [Motor Neuron Disease] explode all trees 1131

#11 (Motor neuron disease or Motor neuron diseases or amyotrophic lateral sclerosis or primary lateral sclerosis or progressive bulbar palsy or progressive muscular atrophy):ti,kw 1758

#12 MeSH descriptor: [Diabetic Neuropathies] explode all trees 3118

#13 (asymmetric diabetic proximal motor neuropath* or diabetic amyotroph* or diabetic asymmetric polyneuropath* or diabetic autonomic neuropath* or diabetic mononeuropath* or diabetic neuralg* or diabetic neuropath* or diabetic polyneuropath* or symmetric diabetic proximal motor neuropath*):ti,kw 4081

#14 MeSH descriptor: [Nervous System Neoplasms] explode all trees 4253

#15 (Nervous System Cancer* or Brain tumor or brain neoplasm* or glioma* or meningioma* or peripheral nerve tumor* spinal cord tumor*):ti,kw 8136

#16 MeSH descriptor: [Alzheimer Disease] this term only 5452

#17 (Alzheimer* disease or dementia or mild cognitive impairment):ti,kw 22974

#18 MeSH descriptor: [Guillain-Barre Syndrome] explode all trees 100

#19 (acute autoimmune neuropathy or acute inflammatory demyelinating or acute inflammatory polyneuropathy or acute inflammatory polyradiculoneuropathy or guillain*barre syndrome):ti,kw 272

#20 MeSH descriptor: [Epilepsy] explode all trees 3529

#21 (aura* or epileps* or seizure disorder*):ti,kw 8428

#22 MeSH descriptor: [Headache] explode all trees 3213

#23 MeSH descriptor: [Headache Disorders] explode all trees 4956

#24 (cephalalgia* or cephalgia* or cephalodynia* or cranial pain* or head pain* or headache* or hemicrania or migraine):ti,kw 35895

#25 MeSH descriptor: [Multiple Sclerosis] explode all trees 5296

#26 (disseminated sclerosis or multiple sclerosis):ti,kw 12027

#27 MeSH descriptor: [Parkinson Disease] explode all trees 6220

#28 (paralysis agitan* or parkinson* disease* or primary parkinsonism):ti,kw 12372

#29 MeSH descriptor: [Stroke] explode all trees 17885

#30 (Stroke or ischemic stroke or intracerebral hemorrhage or subarachnoid hemorrhage or transient ischemic attack):ti,kw 57426

#31 {OR #10-#30} 161216

#32 MeSH descriptor: [Algorithms] explode all trees 8181

#33 MeSH descriptor: [Risk Factors] explode all trees 38474

#34 MeSH descriptor: [Sensitivity and Specificity] explode all trees 21923

#35 (algorithm* or "artificial intelligence" or "clinical decision support" or "machine learning" or informatics or model* or nomogram* or predict* or risk* or "roc curve" or "sensitivity and specificity"):ti,ab,kw 544314

#36 {OR #32-#35} 545623

#37 MeSH descriptor: [Survival Rate] explode all trees 13751

#38 (mortality or survival*):ti,ab,kw 226040

#39 #37 OR #38 226040

#40 MeSH descriptor: [Adult] explode all trees 624990

#41 (adult* or age* or elderly):ti,ab,kw 1375932

#42 #40 OR #41 1375932

#43 #9 AND #31 AND #36 AND #39 AND #42 1365

Cochrane Reviews=115

Cochrane Trials=1263

**References**

1. Hillen T, Davies S, Rudd AG, et al (2003) Self ratings of health predict functional outcome and recurrence free survival after stroke. Journal of Epidemiology & Community Health 57:960–966. https://doi.org/10.1136/jech.57.12.960

2. Walker JD, Maxwell CJ, Hogan DB, Ebly EM (2004) Does Self-Rated Health Predict Survival in Older Persons with Cognitive Impairment? Journal of the American Geriatrics Society 52:1895–1900. https://doi.org/10.1111/j.1532-5415.2004.52515.x

3. Xie J, Brayne C, Matthews FE (2008) Survival times in people with dementia: analysis from population based cohort study with 14 year follow-up. https://doi.org/10.1136/bmj.39433.616678.25

4. Nielsen ABS, Siersma V, Waldemar G, Waldorff FB (2016) Poor self-rated health did not increase risk of permanent nursing placement or mortality in people with mild Alzheimer’s disease. BMC Geriatrics 16:87. https://doi.org/10.1186/s12877-016-0262-x

5. Mavaddat N, Linde R van der, Parker R, et al (2016) Relationship of Self-Rated Health to Stroke Incidence and Mortality in Older Individuals with and without a History of Stroke: A Longitudinal Study of the MRC Cognitive Function and Ageing (CFAS) Population. PLOS ONE 11:e0150178. https://doi.org/10.1371/journal.pone.0150178

6. Araújo É de F, Viana RT, Teixeira-Salmela LF, et al (2019) Self-rated health after stroke: a systematic review of the literature. BMC Neurology 19:221. https://doi.org/10.1186/s12883-019-1448-6

7. González-Vélez AE, Forjaz MJ, Giraldez-García C, et al (2015) Quality of life by proxy and mortality in institutionalized older adults with dementia. Geriatrics & Gerontology International 15:38–44. https://doi.org/10.1111/ggi.12225

8. Phung TKT, Siersma V, Vogel A, et al (2018) Self-rated versus Caregiver-rated Health for Patients with Mild Dementia as Predictors of Patient Mortality. The American Journal of Geriatric Psychiatry 26:375–385. https://doi.org/10.1016/j.jagp.2017.06.005

9. Naess H, Nyland H (2013) Poor health-related quality of life is associated with long-term mortality in young adults with cerebral infarction. J Stroke Cerebrovasc Dis 22:e79-83. https://doi.org/10.1016/j.jstrokecerebrovasdis.2012.06.010

10. del Aguila MA, Longstreth WT, McGuire V, et al (2003) Prognosis in amyotrophic lateral sclerosis. Neurology 60:813–819. https://doi.org/10.1212/01.WNL.0000049472.47709.3B

11. Kielbergerová L, Mayer O, Vaněk J, et al (2015) Quality of life predictors in chronic stable post-stroke patients and prognostic value of SF-36 score as a mortality surrogate. Transl Stroke Res 6:375–383. https://doi.org/10.1007/s12975-015-0418-6

12. Mead GE, Graham C, Dorman P, et al (2011) Fatigue after Stroke: Baseline Predictors and Influence on Survival. Analysis of Data from UK Patients Recruited in the International Stroke Trial. PLOS ONE 6:e16988. https://doi.org/10.1371/journal.pone.0016988

13. Grool AM, van der Graaf Y, Mali WPTM, et al (2012) Mood Problems Increase the Risk of Mortality in Patients With Lacunar Infarcts: the SMART-MR Study. Biopsychosocial Science and Medicine 74:234. https://doi.org/10.1097/PSY.0b013e31824f5ab0

14. Bosma I, Reijneveld JC, Douw L, et al (2009) Health-related quality of life of long-term high-grade glioma survivors. Neuro-Oncology 11:51–58. https://doi.org/10.1215/15228517-2008-049

15. Bell CL, LaCroix A, Masaki K, et al (2013) Prestroke Factors Associated with Poststroke Mortality and Recovery in Older Women in the Women’s Health Initiative. Journal of the American Geriatrics Society 61:1324–1330. https://doi.org/10.1111/jgs.12361

16. Hubbard IJ, Vo K, Forder PM, Byles JE (2016) Stroke, Physical Function, and Death Over a 15-Year Period in Older Australian Women. Stroke 47:1060–1067. https://doi.org/10.1161/STROKEAHA.115.011456

17. Liira H, Mavaddat N, Eineluoto M, et al (2018) Health-related quality of life as a predictor of mortality in heterogeneous samples of older adults. Eur Geriatr Med 9:227–234. https://doi.org/10.1007/s41999-018-0029-3

18. Lou J-S, Moore D, Gordon PH, Miller R (2010) Correlates of quality of life in ALS: Lessons from the minocycline study. Amyotroph Lateral Scler 11:116–121. https://doi.org/10.3109/17482960902918719

19. McCarter H, Furlong W, Whitton AC, et al (2006) Health Status Measurements at Diagnosis As Predictors of Survival Among Adults With Brain Tumors. JCO 24:3636–3643. https://doi.org/10.1200/JCO.2006.06.0137

20. Sturm JW, Osborne RH, Dewey HM, et al (2002) Brief Comprehensive Quality of Life Assessment After Stroke. Stroke 33:2888–2894. https://doi.org/10.1161/01.STR.0000040407.44712.C7

21. Brown PD, Maurer MJ, Rummans TA, et al (2005) A Prospective Study of Quality of Life in Adults with Newly Diagnosed High-grade Gliomas: The Impact of the Extent of Resection on Quality of Life and Survival. Neurosurgery 57:495. https://doi.org/10.1227/01.NEU.0000170562.25335.C7

22. Thakore NJ, Pioro EP (2016) Depression in ALS in a large self-reporting cohort. Neurology 86:1031–1038. https://doi.org/10.1212/WNL.0000000000002465

23. Ayerbe L, Ayis S, Crichton SL, et al (2014) Explanatory factors for the increased mortality of stroke patients with depression. Neurology 83:2007–2012. https://doi.org/10.1212/WNL.0000000000001029

24. Ertel KA, Glymour MM, Glass TA, Berkman LF (2007) Frailty modifies effectiveness of psychosocial intervention in recovery from stroke. Clin Rehabil 21:511–522. https://doi.org/10.1177/0269215507078312

25. Winovich DT, Longstreth WT, Arnold AM, et al (2017) Factors Associated With Ischemic Stroke Survival and Recovery in Older Adults. Stroke 48:1818–1826. https://doi.org/10.1161/STROKEAHA.117.016726

26. Noll KR, Sullaway CM, Wefel JS (2019) Depressive symptoms and executive function in relation to survival in patients with glioblastoma. J Neurooncol 142:183–191. https://doi.org/10.1007/s11060-018-03081-z

27. John F, Michelhaugh SK, Barger GR, et al (2021) Depression and tryptophan metabolism in patients with primary brain tumors: Clinical and molecular imaging correlates. Brain Imaging and Behavior 15:974–985. https://doi.org/10.1007/s11682-020-00305-7

28. Wang S, Li T, Zhou T, et al (2022) Survival in patients with Parkinson’s disease: a ten-year follow-up study in northern China. BMC Neurology 22:367. https://doi.org/10.1186/s12883-022-02899-5

29. Glader E-L, Stegmayr B, Asplund K (2002) Poststroke Fatigue. Stroke 33:1327–1333. https://doi.org/10.1161/01.STR.0000014248.28711.D6

30. Mokhber N, Sheikh Andalibi MS, Morovatdar N, et al (2021) Self-perceived acute psychological stress and risk of mortality, recurrence and disability after stroke: Mashhad Stroke Incidence Study. Stress and Health 37:819–825. https://doi.org/10.1002/smi.3031

31. Ackrivo J, Hansen-Flaschen J, Wileyto EP, et al (2019) Development of a prognostic model of respiratory insufficiency or death in amyotrophic lateral sclerosis. European Respiratory Journal 53:. https://doi.org/10.1183/13993003.02237-2018

32. Thakore NJ, Lapin BR, Kinzy TG, Pioro EP (2018) Deconstructing progression of amyotrophic lateral sclerosis in stages: a Markov modeling approach. Amyotroph Lateral Scler Frontotemporal Degener 19:483–494. https://doi.org/10.1080/21678421.2018.1484925

33. van Eijk RPA, van den Berg LH, Lu Y (2024) Cultivating Patient Preferences in ALS Clinical Trials. Neurology 103:e209502. https://doi.org/10.1212/WNL.0000000000209502

34. Xu Z, Alruwaili ARS, Henderson RD, McCombe PA (2017) Screening for cognitive and behavioural impairment in amyotrophic lateral sclerosis: Frequency of abnormality and effect on survival. Journal of the Neurological Sciences 376:16–23. https://doi.org/10.1016/j.jns.2017.02.061

35. Paillisse C, Lacomblez ,L., Dib ,M., et al (2005) Prognostic factors for survival in amyotrophic lateral sclerosis patients treated with riluzole. Amyotrophic Lateral Sclerosis 6:37–44. https://doi.org/10.1080/14660820510027035

36. Chiò A, Ilardi A, Cammarosano S, et al (2012) Neurobehavioral dysfunction in ALS has a negative effect on outcome and use of PEG and NIV. Neurology 78:1085–1089. https://doi.org/10.1212/WNL.0b013e31824e8f53

37. Paquette B, Vernerey D, Chauffert B, et al (2016) Prognostic value of health-related quality of life for death risk stratification in patients with unresectable glioblastoma. Cancer Med 5:1753–1764. https://doi.org/10.1002/cam4.734

38. Mauer M, Stupp R, Taphoorn MJB, et al (2007) The prognostic value of health-related quality-of-life data in predicting survival in glioblastoma cancer patients: results from an international randomised phase III EORTC Brain Tumour and Radiation Oncology Groups, and NCIC Clinical Trials Group study. Br J Cancer 97:302–307. https://doi.org/10.1038/sj.bjc.6603876

39. Mauer MEL, Taphoorn MJB, Bottomley A, et al (2007) Prognostic value of health-related quality-of-life data in predicting survival in patients with anaplastic oligodendrogliomas, from a phase III EORTC brain cancer group study. J Clin Oncol 25:5731–5737. https://doi.org/10.1200/JCO.2007.11.1476

40. Armstrong TS, Wefel JS, Wang M, et al (2013) Net Clinical Benefit Analysis of Radiation Therapy Oncology Group 0525: A Phase III Trial Comparing Conventional Adjuvant Temozolomide With Dose-Intensive Temozolomide in Patients With Newly Diagnosed Glioblastoma. J Clin Oncol 31:4076–4084. https://doi.org/10.1200/JCO.2013.49.6067

41. Ediebah DE, Galindo-Garre F, Uitdehaag BMJ, et al (2015) Joint modeling of longitudinal health-related quality of life data and survival. Qual Life Res 24:795–804. https://doi.org/10.1007/s11136-014-0821-6

42. Quinten C, Martinelli F, Coens C, et al (2014) A global analysis of multitrial data investigating quality of life and symptoms as prognostic factors for survival in different tumor sites. Cancer 120:302–311. https://doi.org/10.1002/cncr.28382

43. Wefel JS, Armstrong TS, Pugh SL, et al (2021) Neurocognitive, symptom, and health-related quality of life outcomes of a randomized trial of bevacizumab for newly diagnosed glioblastoma (NRG/RTOG 0825). Neuro-Oncology 23:1125–1138. https://doi.org/10.1093/neuonc/noab011

44. Peters KB, West MJ, Hornsby WE, et al (2014) Impact of Health-Related Quality of Life and Fatigue on Survival of Recurrent High-Grade Glioma Patients. J Neurooncol 120:499–506. https://doi.org/10.1007/s11060-014-1574-3

45. Sehlen S, Lenk M, Hollenhorst H, et al (2003) Quality of Life (QoL) as Predictive Mediator Variable for Survival in Patients with Intracerebral Neoplasma During Radiotherapy. Onkologie 26:38–43. https://doi.org/10.1159/000069862

46. Forsaa EB, Larsen JP, Wentzel-Larsen T, Alves G (2010) What predicts mortality in Parkinson disease? Neurology 75:1270–1276. https://doi.org/10.1212/WNL.0b013e3181f61311

47. Krishnan S, Pisharady KK, Rajan R, et al (2019) Predictors of dementia-free survival after bilateral subthalamic deep brain stimulation for Parkinson’s disease. Neurology India 67:459. https://doi.org/10.4103/0028-3886.258056

48. Raffel J, Wallace A, Gveric D, et al (2017) Patient-reported outcomes and survival in multiple sclerosis: A 10-year retrospective cohort study using the Multiple Sclerosis Impact Scale–29. PLOS Medicine 14:e1002346. https://doi.org/10.1371/journal.pmed.1002346

49. Zietemann V, Georgakis MK, Dondaine T, et al (2018) Early MoCA predicts long-term cognitive and functional outcome and mortality after stroke. Neurology 91:e1838–e1850. https://doi.org/10.1212/WNL.0000000000006506
